# Experiment-based computational model predicts that IL-6 classic and trans-signaling exhibit similar potency in inducing downstream signaling in endothelial cells

**DOI:** 10.1038/s41540-023-00308-2

**Published:** 2023-09-21

**Authors:** Min Song, Youli Wang, Brian H. Annex, Aleksander S. Popel

**Affiliations:** 1 Department of Biomedical Engineering, Johns Hopkins University School of Medicine, Baltimore, MD 21205 USA; 2 Department of Medicine, Augusta University Medical College of Georgia, Augusta, GA 30912 USA

**Keywords:** Systems biology, Computational biology and bioinformatics

## Abstract

Inflammatory cytokine mediated responses are important in the development of many diseases that are associated with angiogenesis. Targeting angiogenesis as a prominent strategy has shown limited effects in many contexts such as cardiovascular diseases and cancer. One potential reason for the unsuccessful outcome is the mutual dependent role between inflammation and angiogenesis. Inflammation-based therapies primarily target inflammatory cytokines such as interleukin-6 (IL-6) in T cells, macrophages, cancer cells, and muscle cells, and there is a limited understanding of how these cytokines act on endothelial cells. Thus, we focus on one of the major inflammatory cytokines, IL-6, mediated intracellular signaling in endothelial cells by developing a detailed computational model. Our model quantitatively characterized the effects of IL-6 classic and trans-signaling in activating the signal transducer and activator of transcription 3 (STAT3), phosphatidylinositol 3-kinase/protein kinase B (PI3K/Akt), and mitogen-activated protein kinase (MAPK) signaling to phosphorylate STAT3, extracellular regulated kinase (ERK) and Akt, respectively. We applied the trained and validated experiment-based computational model to characterize the dynamics of phosphorylated STAT3 (pSTAT3), Akt (pAkt), and ERK (pERK) in response to IL-6 classic and/or trans-signaling. The model predicts that IL-6 classic and trans-signaling induced responses are IL-6 and soluble IL-6 receptor (sIL-6R) dose-dependent. Also, IL-6 classic and trans-signaling showed similar potency in inducing downstream signaling; however, trans-signaling induces stronger downstream responses and plays a dominant role in the overall effects from IL-6 due to the in vitro experimental setting of abundant sIL-6R. In addition, both IL-6 and sIL-6R levels regulate signaling strength. Moreover, our model identifies the influential species and kinetic parameters that specifically modulate the downstream inflammatory and/or angiogenic signals, pSTAT3, pAkt, and pERK responses. Overall, the model predicts the effects of IL-6 classic and/or trans-signaling stimulation quantitatively and provides a framework for analyzing and integrating experimental data. More broadly, this model can be utilized to identify potential targets that influence IL-6 mediated signaling in endothelial cells and to study their effects quantitatively in modulating STAT3, Akt, and ERK activation.

## Introduction

Angiogenesis is the formation of new blood capillaries from pre-existing blood vessels^1^. Inflammatory cytokine mediated responses and angiogenesis play an important role in many diseases, such as cardiovascular diseases^2,3^, cancer^1,4^, and ocular diseases^5–7^, as well as regenerative medicine^8,9^ and tissue engineering^10,11^. The essential role of blood vessels in delivering nutrients makes angiogenesis important in the survival of cells within tissues, including tumor growth. Targeting angiogenesis is an important strategy in many contexts, for example, tissue engineering^12^ and cancer treatment^13^; however, it has not always been successful^14–16^. At least one potential explanation for the ineffectiveness of modulating angiogenesis is that this process triggers severe inflammatory responses^17,18^. Specifically, endothelial cells in response to inflammatory cytokines, such as IL-6, get activated and lead to increased vascular leakage, leukocyte recruitment, and further accumulation of plaques, which blocks blood flow^19,20^. On the other hand, inflammation can promote angiogenesis in many ways as well. Specifically, inflammatory tissues are often hypoxic which induces angiogenesis^21^. Also, cells involved in inflammatory processes such as macrophages and fibroblasts secrete angiogenic factors that promote vessel formation^21^. In addition, there is evidence that pro-inflammatory cytokines such as interleukin-6 (IL-6) and tumor necrosis factor alpha (TNFα) promote angiogenesis^21–23^. Thus, inflammation is often associated with angiogenesis^21^ and it plays an important role in the development of many diseases, such as cancer and cardiovascular diseases. Thus, the goal of this study is to investigate, in mechanistic detail using a computational model, IL-6 mediated signaling in vascular endothelial cells.

Numerous experimental and computational studies have investigated the array of responses to inflammatory cytokines in different cell types such as macrophages^24,25^, T cells^26,27^, and cancer cells^28,29^. Also, recent reviews focused on computational models and analysis of angiogenic signaling^30,31^. However, there is limited quantitative analysis of inflammatory together with angiogenic responses in endothelial cells to inform potential treatments that target inflammation and angiogenesis. Therefore, we aim to focus on inflammatory signaling in endothelial cells to characterize endothelial inflammatory and angiogenic responses. The role of many circulating biomarkers, such as selectins and interleukins in peripheral arterial disease has been reviewed^19,32^. Also, potential anti-inflammatory strategies are reviewed for cardiovascular disease^33,34^. In this study, we will focus on the intracellular signaling mediated by one of the major inflammatory cytokines, IL-6, as it has been identified as an important biomarker in inflammation in many diseases such as cardiovascular disease and cancer^19,32,33,35^. In addition, elevated levels of IL-6^36–41^ and soluble IL-6 receptors (sIL-6R)^41,42^ have been demonstrated in pathological conditions, including peripheral arterial disease and cancer.

Interestingly, IL-6 can act as a both pro- and anti-inflammatory factor^35^. IL-6 signaling transduces via binding to its membrane bound receptor (IL-6R) is referred to as classic signaling. When IL-6 binds to its soluble receptor sIL-6R, and then recruits glycoprotein 130 (gp130) and initiates downstream signaling, this is referred to as trans-signaling^35^. It has been shown that IL-6 classic signaling is associated with anti-inflammatory and regenerative responses, while IL-6 trans-signaling is involved in pro-inflammatory responses^35,43^. Specifically, IL-6 binds to its receptors (IL-6R and/or sIL-6R) and gp130 and initiates signaling through the signal transducer and activator of transcription 3 (STAT3), mitogen-activated protein kinase (MAPK) and phosphatidylinositol 3-kinase/protein kinase B (PI3K/Akt) pathways to phosphorylate STAT3, extracellular regulated kinase (ERK) and Akt, respectively. The phosphorylated STAT3 (pSTAT3) and Akt (pAkt) are important signaling species in the inflammatory responses^44^, while pAkt is believed to play an important role in cell survival^45–49^ and phosphorylated ERK (pERK) is critical in cell proliferation^50,51^, which are important processes involved in angiogenesis. Thus, we mainly focus on IL-6 trans-signaling mediated pSTAT3 and pAkt responses as indicators for pro-inflammatory signaling, and IL-6 classic signaling mediated Akt and ERK activation as signaling species for pro-angiogenic responses.

Given the complexity of biochemical reactions comprising inflammatory signaling networks, a better understanding of the dynamics of these networks quantitatively is beneficial for current anti-inflammatory strategies targeting endothelial cells. Computational modeling serves as a powerful tool to investigate molecular responses systematically. For example, Reeh et al. developed a mathematical model to investigate IL-6 trans- and classic signaling in human hepatoma cells on a molecular level^52^. In addition, Mitchell et al. constructed a mathematical model of nuclear factor kappa B (NF-κB) activity and investigated the effects of interferons^53^. Furthermore, Zhao et al. developed a large-scale mechanistic model which focused on seven driving pathways including interferon gamma (IFNγ), IL-1β, IL-10, IL-4, TNFα, hypoxia, and VEGF to characterize macrophage polarization^54^. Later, Zhao et al. constructed a multiscale model that considers inflammatory signaling and includes intracellular, cellular, and tissue-level features to study the dynamic reconstitution of perfusion during post hindlimb ischemia^55^.

Therefore, we constructed a computational model to characterize the intracellular signaling mediated by IL-6 in endothelial cells. Our work is the first model that focuses on IL-6 mediated signaling in endothelial cells to characterize endothelial inflammatory and angiogenic responses. The model predicts the dynamics of pSTAT3, pAkt, and pERK in response to IL-6 classic and/or trans-signaling. The model predicts that IL-6 classic and trans-signaling show similar potency in inducing downstream responses. However, due to the in vitro experimental setting of abundant sIL-6R, IL-6 trans-signaling induces stronger downstream signaling and promotes inflammatory responses, and it plays a dominate role in the overall effects under this condition. In addition, both IL-6 and sIL-6R levels regulate signaling strength. Using this model, we also identified the influential species and kinetic parameters that specifically modulate the downstream inflammatory and/or angiogenic signals, pSTAT3, pAkt, and pERK responses, and investigated their efficacy. The model predictions provide mechanistic insight into IL-6 signaling in endothelial cells. More broadly, this model provides a framework to study the efficacy of inflammation- and angiogenesis-based therapies for endothelial cells.

## Results

### The fitted and validated molecular-detailed computational model captures the major characteristics of IL-6 induced STAT3, Akt, and ERK phosphorylation dynamics

**Fig. 1:**
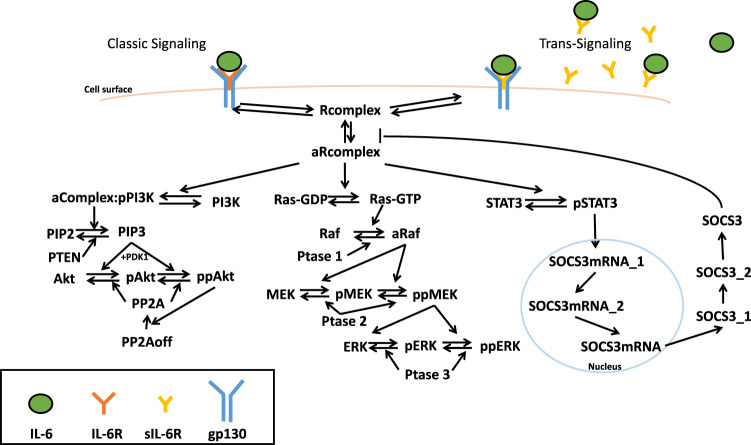
Schematic of IL-6 signaling network. IL-6 classic and trans-signaling is induced by IL-6 binding to membrane-bound and soluble IL-6 receptors, respectively, and recruiting gp130, which activates PI3K/Akt, MAPK, and STAT3 pathways and phosphorylates Akt, ERK, and STAT3, respectively.

For model training, we first identified the model variables (kinetic rates, initial concentrations, and factor ratio4) that significantly influence the model outputs, pSTAT3, pAkt, and pERK (Fig. 1). To do so, we performed a sensitivity analysis using PRCC (see Methods for more details) and analyzed the PRCC values for all the species concentrations and kinetic rates. The highest PRCC values across all of the outputs and time points for a total of 65 species, 68 parameters, and 1 factor that affect initial concentrations, were compared, and 35 of them (Supplementary Table 4 and Supplementary Fig. 1) were identified as influential to pSTAT3, pAkt, and pERK induced by 0–50 ng/ml IL-6 alone and/or with additional 100 ng/ml sIL-6R, which are the same concentrations applied experimentally^52^. Of these, 28 of them were not correlated (highlighted in red, Supplementary Table 4 and Supplementary Fig. 2), and we then estimated their values by fitting the model to experimental measurements^52^ using PSO^56^ (see Methods for more details).

**Fig. 2:**
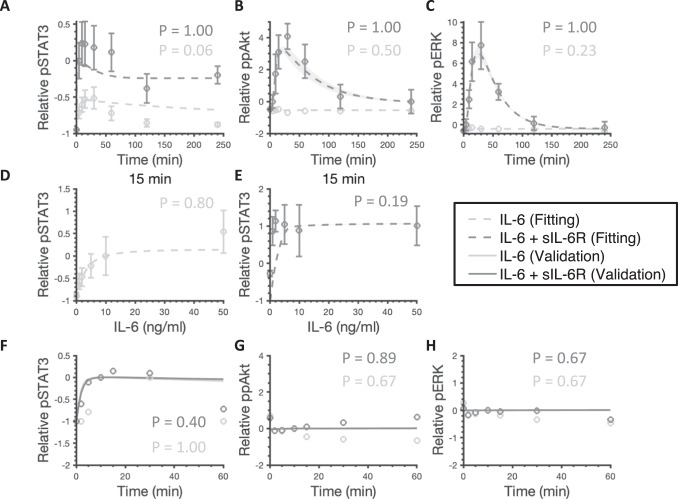
Model comparison to training and validation data for IL-6 stimulation. 50 ng/ml IL-6 with or without additional 100 ng/ml sIL-6R induced relative pSTAT3 (**A**), ppAkt (**B**), and pERK (**C**). Varying concentrations of IL-6 alone induced relative pSTAT3 (**D**) and with additional 100 ng/ml sIL-6R induced relative pSTAT3 (**E**). The circles are experimental data. Bars are mean ± SEM. Curves are the mean values of the 12 best fits. Shaded regions show 95% confidence intervals of the fits. Dashed and solid curves are training and validation results, respectively. Light gray: 50 ng/ml IL-6 (**A**–**C**), 0–50 ng/ml IL-6 (**D**), and 10 ng/ml IL-6 (**F**–**H**) stimulation; Dark gray: 50 ng/ml IL-6 + 100 ng/ml sIL-6R (**A**–**C**), 0–50 ng/ml IL-6 + 100 ng/ml sIL-6R (**E**), and 10 ng/ml IL-6 + 10 ng/ml sIL-6R (**F**–**H**).

The fitted model shows a good agreement with experimental results (Fig. 2A–E). It quantitatively captures the dynamics of pSTAT3, ppAkt, and pERK by the stimulation of 50 ng/ml IL-6 alone (Fig. 2A–C, light gray) and in combination with an additional 100 ng/ml sIL-6R (Fig. 2A–C, dark gray)^44^. In addition, varying concentrations of IL-6 alone (Fig. 2D) and in combination with an additional 100 ng/ml sIL-6R (Fig. 2E) induced-pSTAT3 dose responses have a good agreement with experimental measurements^44^.

In addition to model fitting, the model predictions are consistent with independent experimental observations that are not used in the model training (Fig. 2F–H). To validate the model, we compared the model predictions to three independent sets of experimental data^57^. Specifically, STAT3, Akt, and ERK phosphorylation by the stimulation of 10 ng/ml IL-6 alone (Fig. 2F–H, light gray) and in combination with 10 ng/ml sIL-6R (Fig. 2F–H, dark gray) matched the additional experimental measurements^57^. A total of 12 parameter sets were taken to be the “best” sets based on the model fitting (Fig. 2A–E) and validation (Fig. 2F–H) and were used for all model simulations; the smallest weighted errors ranged from 26.53 to 33.80 and *p*-values greater than 0.05 by performing the runs test (Supplementary Table 6).

It is noteworthy that we compared the predicted doubly phosphorylated Akt (ppAkt) to experimental data for model fitting and validation^44,57^ (see Methods for more details). However, since both Akt T308 and S473 phosphorylation have been shown to play an important role in the downstream signaling^58^, we considered both singly and doubly phosphorylated forms of Akt to study its activation in the remainder of this work.

We performed Monte Carlo simulations (see Methods for more details) to study the predicted pSTAT3, pAkt, and pERK levels given the variability in the initial concentrations and parameters. The model predictions with parameter values randomly varied within the range of the estimated values can still capture pSTAT3, pAkt, and pERK dynamics stimulated by IL-6 alone and in combination with sIL-6R (Supplementary Fig. 3). These simulations suggest that the overall dynamics of the model outputs, pSTAT3, pAkt, and pERK, are relatively robust to variability or uncertainty in initial concentrations and parameters in the signaling network.

### No activation of ERK was observed in response to IL-6 stimulation alone and IL-6 classic and trans-signaling induced responses are dose-dependent

**Fig. 3:**
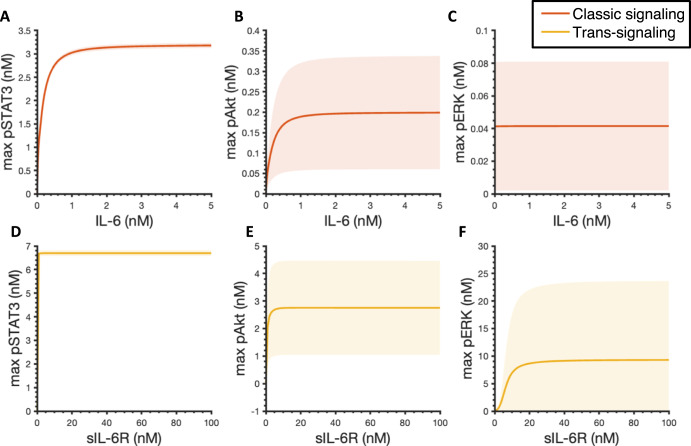
Predicted maximum pSTAT3, pAkt, and pERK responses. Maximum pSTAT3 (**A**), pAkt (**B**), and pERK (**C**) in response to IL-6 concentrations varying from 0 to 5 nM without sIL-6R. In the absence of IL-6R, 2 nM IL-6 in combination with sIL-6R concentrations varying from 0 to 100 nM induced maximum pSTAT3 (**D**), pAkt (**E**), and pERK (**F**). Curves are the mean values of the 12 best fits. Shaded regions show 95% confidence intervals of the fits. Orange: classic signaling responses; Yellow: trans-signaling responses.

We first applied the experimentally validated model to explore the effects of IL-6 classic and trans-signaling on STAT3, Akt, and ERK phosphorylation. We found that the maximum pSTAT3 and pAkt levels within four hours increase with the increase of IL-6 concentrations (Fig. 3A, B). IL-6-induced pSTAT3 and pAkt exhibit optimal ligand levels for inducing maximum responses as their dose response plateaus approximately at 2 nM by the stimulation of ligand concentration in the range of 0 nM – 5 nM (Fig. 3A, B). It is noteworthy that no activation of ERK was observed in response to IL-6 stimulation alone. In addition, the area under the curve (AUC) is quantified for pSTAT3, pAkt, and pERK dynamics within four hours as well and they exhibit a similar behavior (Supplementary Fig. 4A–C).

We then set IL-6R level to zero and simulated the phosphorylation of STAT3, Akt, and ERK in response to the stimulation of 2 nM IL-6 in combination with varying concentrations of sIL-6R to study the effects of IL-6 trans-signaling. A dose-dependent manner of STAT3, Akt, and ERK activation is also observed when considering the maximal phosphorylation levels (Fig. 3D–F) and AUC (Supplementary Fig. 4D–F), respectively. Specifically, the maximum pSTAT3, pAkt, and pERK levels and AUCs increase and plateau with the increase of sIL-6R concentrations within 100 nM sIL-6R in combination with 2 nM IL-6 (Fig. 3D–F and Supplementary Fig. 4D–F).

In addition, since the maximum levels and AUCs exhibit the same trends as we observed (Fig. 3 and Supplementary Fig. 4), for simplification, the maximum pSTAT3, pAkt, and pERK levels within four hours are utilized as indicators for pSTAT3, pERK, and pAkt responses in this study.

### IL-6 classic and trans-signaling exhibited similar potency in inducing downstream responses

**Fig. 4:**
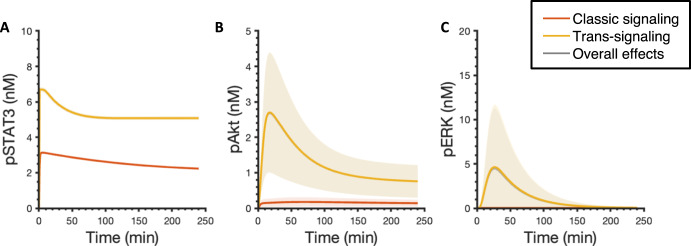
Predicted pSTAT3, pAkt, and pERK time course responses. Predicted time courses of pSTAT3 (**A**), pAkt (**B**), and pERK (**C**) following stimulation by 2 nM IL-6 alone with a mean value of 6.4 nM IL-6R (orange), 2 nM IL-6 in combination with a mean value of 6.4 nM sIL-6R in the absence of IL-6R (yellow), and 2 nM IL-6 with a mean value of 6.4 nM of both IL-6R and sIL-6R (gray). Curves are the mean values of the 12 best fits. Shaded regions show 95% confidence intervals of the fits. Orange: classic signaling responses; Yellow: trans-signaling responses; Gray: overall responses.

To compare the effects of classic and trans-signaling on STAT3, Akt, and ERK phosphorylation, we next set the concentration of sIL-6R at the same level as IL-6R for each fit, which is 6.4 nM on average among the 12 best fits, and simulated the dynamics of pSTAT3, pAkt, and pERK upon the stimulation of 2 nM IL-6 alone in the presence of IL-6R (orange) and 2 nM IL-6 in combination with a mean value of 6.4 nM sIL-6R in the absence of IL-6R (yellow) (Fig. 4). Since IL-6R and sIL-6R are both present in the physiological and pathological conditions, we also studied the overall effects of the stimulation of 2 nM IL-6 in combination with 6.4 nM sIL-6R (mean) in the presence of IL-6R in pSTAT3, pAkt, and pERK responses (Fig. 4, gray curves). We found that the IL-6 trans-signaling induced max pSTAT3 and pAkt and overall effects induced max pSTAT3, pAkt, and pERK are statistically significantly higher than the classic signaling induced corresponding responses, respectively (*p* < 0.05) (Fig. 4, Supplementary Table 7). Also, IL-6 trans-signaling plays a dominant role in the overall effects in inducing pSTAT3, pAkt, and pERK as the overall effects induced responses overlap with the responses induced by the IL-6 trans-signaling (Fig. 4).

To mechanistically explain this phenomenon, we explored the model structure and found that it is mainly caused by an assumption of a constant sIL-6R as our model input since the abundant supply of sIL-6 is added to the cell culture media in the in vitro experimental condition. A similar assumption was also made in Reeh’s model, specifically, Hyper-IL-6, which is a fusion protein composed of sIL-6R and IL-6 was applied to study the effects of IL-6 trans-signaling and it was assumed to be a constant model input as its concentration remained the same in the supernatant experimentally in an in vitro study^52^. A sustained supply of soluble receptors leads to greater downstream responses compared to IL-6 classic signaling as the amount of IL-6R is limited. To verify this hypothesis, we set IL-6R as a constant input, which is the same as sIL-6R, in our model and compared the effects of classic and trans-signaling. We found that pSTAT3, pAkt, and pERK induced by IL-6 classic and trans-signaling almost overlap when IL-6R and sIL-6R are set at the same level and remain constant within four-hour simulation time (Supplementary Fig. 5A and Table 1), which confirms our hypothesis that stronger downstream responses induced by IL-6 trans-signaling are mainly caused by the sustained supply of sIL-6R.

**Table 1:** Predicted maximum pSTAT3, pAkt, and pERK responses for the baseline model, the modified model when IL-6R was set as a constant input, when kinetic rates governing R1 and R2 to be the same as the corresponding kinetic rates for R3 and R4, and when both IL-6R was set as a constant input and kinetic rates governing R1 and R2 to be the same as the corresponding kinetic rates for R3 and R4.

		Max pSTAT3	Max pAkt	Max pERK
Baseline	Classic	3.14	0.18	0.04
	Trans	6.70	2.70	4.64
	Overall	6.70	2.69	4.48
Receptors	Classic	6.69	2.43	0.95
	Trans	6.70	2.70	4.64
	Overall	6.70	2.69	5.67
Params	Classic	3.19	0.18	0.04
	Trans	6.70	2.70	4.64
	Overall	6.70	2.70	4.75
Params & Receptors	Classic	6.70	2.70	4.64
	Trans	6.70	2.70	4.64
	Overall	6.70	2.73	7.59

The units in the table are nM. R1: IL-6 + IL-6R $\longleftrightarrow$ IL-6:IL-6R; R2: 2 IL-6:IL-6R + 2 gp130 $\longleftrightarrow$ Rcomplex; R3: IL-6 + sIL-6R $\longleftrightarrow$ IL-6:sIL-6R; R4: 2 IL-6:sIL-6R + 2 gp130 $\longleftrightarrow$ Rcomplex.

**Fig. 5:**
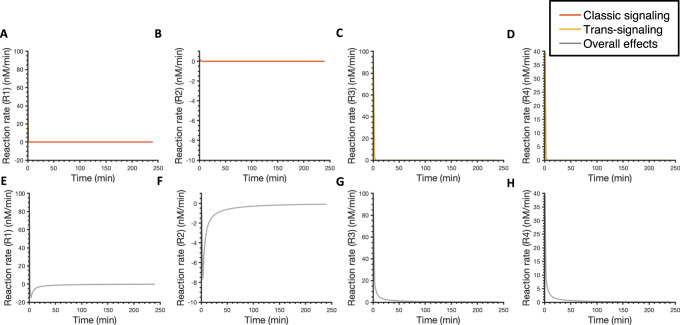
Predicted reaction rates for ligand-receptor binding interactions. Reaction rates for ligand-receptor binding following stimulation by 2 nM IL-6 alone with a mean value of 6.4 nM IL-6R (orange) (**A**, **B**), 2 nM IL-6 in combination with a mean value of 6.4 nM sIL-6R in the absence of IL-6R (yellow) (**C**, **D**), and 2 nM IL-6 with a mean value of 6.4 nM of both IL-6R and sIL-6R (gray) (E-H). R1: IL-6 + IL-6R $\longleftrightarrow$ IL-6:IL-6R; R2: 2 IL-6:IL-6R + 2 gp130 $\longleftrightarrow$ Rcomplex; R3: IL-6 + sIL-6R $\longleftrightarrow$ IL-6:sIL-6R; R4: 2 IL-6:sIL-6R + 2 gp130 $\longleftrightarrow$ Rcomplex. Curves are the mean values of the 12 best fits. Shaded regions show 95% confidence intervals of the fits. Orange: classic signaling responses; Yellow: trans-signaling responses; Gray: overall responses.

We also noticed some differences in the dissociation constant (Kd) for the ligand-receptor binding reactions induced by the IL-6 classic and trans-signaling. Specifically, the Kd for reaction 1 (R1: IL-6 + IL-6R $\longleftrightarrow$ IL-6:IL-6R; mean Kd = 479.6 nM) is lower than the Kd for reaction 3 (R3: IL-6 + sIL-6R $\longleftrightarrow$ IL-6:sIL-6R; Kd = 17.9 nM); while the Kd for reaction 2 (R2: 2 IL-6:IL-6R + 2 gp130 $\longleftrightarrow$ Rcomplex; Kd = 0.05 nM) is higher compared to the Kd for reaction 4 (R4: 2 IL-6:sIL-6R + 2 gp130 $\longleftrightarrow$ Rcomplex; Kd = 0.02 nM) (Supplementary Fig. 6). It suggests a tighter binding of IL-6 to sIL-6R and then gp130 than IL-6R and then gp130. However, no obvious difference was observed in pSTAT3, pAkt, and pERK when we set the kinetic rates governing the ligand-receptor binding reactions for classic signaling (R1 and R2) to be the same as the corresponding kinetic rates for the ligand-receptor binding reactions for trans-signaling (R3 and R4) compared with baseline model predictions (Supplementary Fig. 5B, Fig. 5, and Table 1). It indicates that although there are some differences in ligand-receptor binding reactions induced by the IL-6 classic and trans-signaling, specifically IL-6 binds tighter to sIL-6R and IL-6:sIL-6R binds tighter to gp130 compared to classic signaling, it shows no obvious effects in the downstream signaling: 1.6% increase in max pSTAT3 compared to the baseline model predictions (Table 1).

Last, we set IL-6R and sIL-6R at the same level and they remain constant within four hours and kinetic rates governing R1 and R2 to be the same as the corresponding kinetic rates for R3 and R4 and predicted the dynamics of pSTAT3, pAkt, and pERK (Supplementary Fig. 5C). The activation of STAT3, Akt, and ERK induced by IL-6 classic was found to overlap with the corresponding responses induced by IL-6 trans-signaling.

Overall, the model suggests that IL-6 trans-signaling induces stronger responses than classic signaling, and it plays a dominant role in the overall effects. This is mainly due to the sustained supply of sIL-6R. Thus, it indicates that IL-6 classic and trans-signaling have similar potency in inducing downstream signaling, pSTAT3, pAkt, and pERK; however, the in vitro experimental condition of abundant sIL-6R leads to stronger activation in STAT3, Akt, and ERK induced by IL-6 trans-signaling.

### sIL-6R enhances the downstream signaling and promotes inflammatory responses

We next compared reaction rates for reactions 1–4 with or without IL-6R and sIL-6R (Fig. 5). We found that IL-6 binds to IL-6R as fast as to sIL-6R faster in the beginning as the highest reaction rate for R3 is not statistically different than the highest reaction rate for R1 (*P* > 0.05) (Fig. 5A, C, Supplementary Table 8). However, IL-6:sIL-6R binds faster to gp130 than IL-6:sIL-6R as the highest reaction rate for R4 is statistically higher than the highest reaction rate for R2 in the beginning (*P* < 0.05) (Fig. 5B, D, Supplementary Table 8). It is noteworthy that additional sIL-6R makes reaction rates for R1 and R2 become negative over time (Fig. 5A, B and E, F), which suggests a faster dissociation of IL6:IL6R and Rcomplex compared to the association of IL-6, IL-6R, and gp130. It indicates that more IL-6 and gp130 are freed from binding to IL-6R and becoming available for binding to sIL-6R and inducing trans-signaling. Also, reaction rates for R3 and R4 are more sustained when both IL-6R and sIL-6R are present compared to the reaction rates for trans-signaling (Fig. 5C, D and G, H) since more IL-6 are released from classic signaling. Together, it indicates that additional sIL-6R shifts the signaling towards trans-signaling, which promotes inflammatory responses and this is consistent with the dominant role of trans-signaling in the overall effects.

To further study the model details, we compared the time courses of relevant species involved in R1-R4 with or without IL-6R and sIL-6R (Supplementary Fig. 7). The model predicts that there is more IL-6:sIL-6R formed compared to IL-6:IL-6R (Supplementary Fig. 5A, C). Also, the predicted level of signaling Rcomplex induced by trans-signaling is higher than the classic signaling (Supplementary Fig. 5B, D). These model predictions further confirm that IL-6 trans-signaling induces stronger responses than classic signaling. Moreover, an accumulation of IL-6:IL-6R and a higher consumption of IL-6:sIL-6R induced by the overall effects compared to classic and trans-signaling respectively are observed (Supplementary Fig. 5A compared to E, and C compared to F). It is consistent with our predictions that additional sIL-6R shifts the signaling towards trans-signaling. In addition, the Rcomplex induced by the overall effects is approximately the same level as the Rcomplex induced by trans-signaling (Supplementary Fig. 5D compared to G), which agrees with the dominant role of trans-signaling in the overall effects.

Generally, the model suggests that under the condition of abundant sIL-6R, IL-6 trans-signaling induces stronger responses and additional sIL-6R shifts the signaling towards trans-signaling, which promotes pro-inflammatory responses.

### Both IL-6 and sIL-6R levels regulate signaling strength

**Fig. 6:**
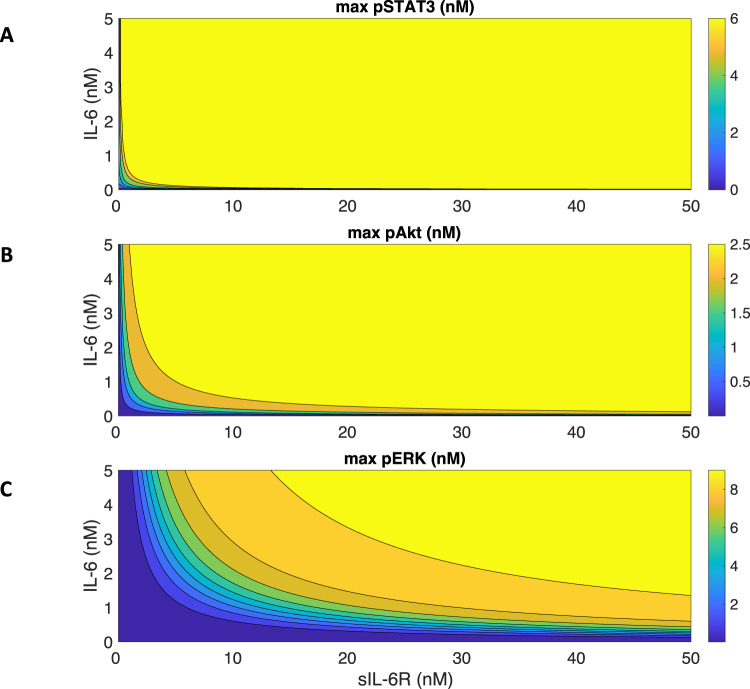
Predicted maximum pSTAT3, pAkt, and pERK responses with varying concentrations of IL-6 and sIL-6R. Maximum pSTAT3 (**A**), pAkt (**B**), and pERK (**C**) in response to the stimulation of 0–5 nM IL-6 in combination with 0–50 nM sIL-6R.

We next varied IL-6 and sIL-6R simultaneously and studied their combination effects in STAT3, Akt, and ERK activation. We found that there is a gradient towards the diagonal direction of increasing IL-6 and sIL-6R concentrations for each signaling species (Fig. 6). As we observed previously, STAT3, Akt, and ERK activation plateau at approximately 2 nM IL-6 stimulation (Fig. 3A–C), while additional sIL-6R further promotes the downstream signaling (Fig. 6). Also, at a certain level of sIL-6R, adding IL-6 increases the STAT3, Akt, and ERK activation as well (Fig. 6). An upregulation of IL-6^39^ and sIL-6R^59^ has been reported in the peripheral arterial disease conditions, which leads to stronger inflammatory responses. It is consistent with our model predictions as higher IL-6R and sIL-6R levels lead to greater phosphorylation of STAT3, Akt, and ERK (Fig. 6).

### Model identifies potential targets for influencing STAT3, Akt, and ERK activation and quantitively evaluates their efficacy

We performed a sensitivity analysis using PRCC (see Methods for more details) for the experimentally validated model and identified influential initial concentrations (Supplementary Fig. 8A–C) and parameters (Supplementary Fig. 8D, E) to STAT3, Akt, and ERK activation. Specifically, all model parameters and initial values were sampled within two orders of magnitude above and two orders of magnitude below the baseline values. In this case, the baseline values for the fitted variables were the best fit estimated from model fitting. Based on the behaviors of max pSTAT3, pAkt, and pERK that reach a plateau as the IL-6 concentration increases (Fig. 3), we selected 2 nM IL-6 as a representative concentration to capture the optimal responses induced by classic signaling. Also, to compare the effects of IL-6 classic and trans-signaling, we took 6.3 nM sIL-6 as a representative concentration since it is the same level as the IL-6R concentration from the best fit. Therefore, we calculated the PRCC values for pSTAT3, pAkt, and pERK in response to the stimulation of 2 nM IL-6 in combination of 6.3 nM sIL-6R at eight time points (0, 5, 10, 15, 30, 60, 120, and 240 min) ranging from zero to 240 min. Again, the *PRCC*_*max*_ across all the concentrations and time points was compared for all the variables.

**Fig. 7:**
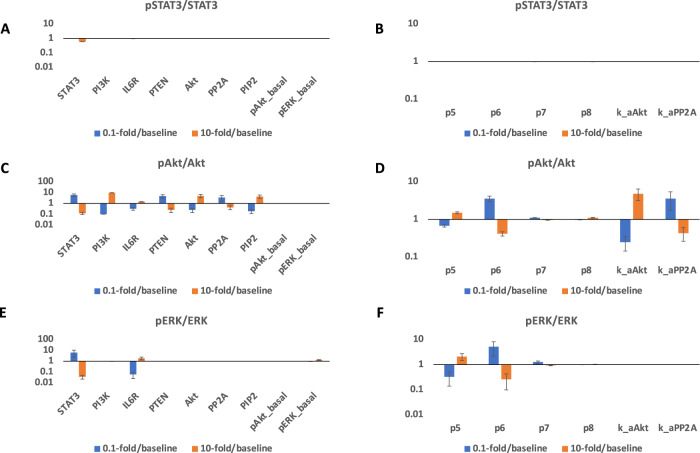
Predicted targets for modulating pSTAT3, pAkt, and pERK responses. 0.1-fold/baseline (blue) and 10-fold/baseline (orange) for 2 nM IL-6 with a mean value of 6.4 nM of IL-6R and 6.4 nM sIL-6R induced pSTAT3/total STAT3 (**A**, **D**), pAkt/total Akt (**B**, **E**), and pERK/total ERK (**C**, **F**) when varying identified influential initial concentrations (left) and parameters (right) by 0.1- and 10-fold of their baseline values. Bars are mean ± 95% confidence intervals of model predictions.

To analyze their effects in pSTAT3, pAkt, and pERK quantitatively, we varied each of identified influential variables within a finite range, specifically 10-fold above and below the baseline levels and compared with the baseline model predictions (Fig. 7). When the ratio is greater than one, it suggests that varying the variable promotes the response; when the ratio is equal to one, it shows no effects on the response; when the ratio is less than one, it indicates an inhibitory effect on the response. We consider the effects of the perturbations as effective when the change of response is greater than 2-fold or less than 0.5-fold. We found that no initial concentration or parameter was observed to influence pSTAT3/STAT3 significantly (Fig. 7A, B). In addition, Akt phosphorylation is positively regulated by PI3K, IL-6R, Akt, and PIP2 levels (Fig. 7C); while it is negatively regulated by STAT3, PTEN, and PP2A levels (Fig. 7C). This is intuitive as PI3K, IL-6R, Akt, and PIP2 are important signaling upstream species for Akt phosphorylation. PTEN and PP2A are phosphatases for PIP3 and pAkt. The impact of STAT3 level in the Akt phosphorylation is due to the competition between STAT3 signaling and the Akt pathway. Also, parameter k_aAkt positively regulates pAkt, while p6 and k_aPP2A negatively regulate pAkt (Fig. 7D) as k_aAkt is the association rate of PIP3 and Akt/pAkt, p6 is the deactivation rate pf Rcomplex, and k_aPP2A is the association rate of pAkt/ppAkt and PP2A. Last, IL-6R positively regulates pERK, while STAT3 negatively regulates ERK phosphorylation (Fig. 7E). Because IL-6R is an upstream species for ERK phosphorylation and STAT3 is a signaling species involved in the competitive pathways and negatively influences ERK activation. Also, parameter p5 positively regulates pERK, while p6 negatively regulates it (Fig. 7F) as p5 and p6 are the activation and deactivation rate of Rcomplex.

Thus, our model identifies potential targets for modulating downstream inflammatory and/or angiogenic signals, pSTAT3, pAkt, and pERK in response to the overall effects of IL-6 classic and trans-signaling and quantitively evaluates their efficacy.

## Discussion

We developed an intracellular signaling model of IL-6 mediated inflammatory pathways in endothelial cells. The detailed computational model represents the reaction network of interactions on a molecular level. The model includes molecular interactions, kinetic parameters, and initial concentrations documented in the literature, which are provided in the supplementary materials (Supplementary Table 1–3). Influential parameters were estimated by fitting the model to experimental data^44^. Additionally, we validated the model using three independent experimental datasets^44^. All experimental data we used in model training and validation are in vitro HUVEC data. Thus, this model is constructed specifically to characterize in vitro HUVEC responses and could be used as the foundation to model in vivo experiments in the future.

IL-6 classic signaling is believed to be associated with anti-inflammatory or regenerative responses, while IL-6 trans-signaling is important in pro-inflammatory responses^43^. It has been reported that IL-6 trans-signaling induces monocyte chemoattractant protein-1 (MCP-1) expression via activating STAT3 and Akt pathways, but not MAPK signaling in HUVECs^44^. Also, ERK activation is mainly believed to be important in cell proliferation^50^. PI3K/Akt pathway has been reported to be critical in regulating cell survival and migration^45–49,60,61^. Therefore, pSTAT3, pAkt, and pERK are the main indices for inflammation and angiogenesis in this study. Specifically, this model focuses on IL-6 trans-signaling mediated pSTAT3 and pAkt responses as indicators for pro-inflammatory signaling, and IL-6 classic signaling mediated Akt and ERK activation as signaling species for pro-angiogenic responses.

The fitted model predicts pSTAT3, pAkt, and pERK responses upon the stimulation by IL-6 classic and/or trans-signaling. Overall, the model suggests that the max pSTAT3, pAkt, and pERK levels are IL-6 and sIL-6R dose-dependent. It has been shown that STAT3 phosphorylation in response to IL-6 classic and trans-signaling is dose-dependent in human hepatoma cells (HepG2)^52^ and endothelial cells^44^, which is consistent with our model predictions. Also, slight activation of Akt and no obvious activation of ERK was observed in response to IL-6 stimulation alone (Fig. 3B–C). This is also consistent with the experimental observation of no obvious activation of Akt and ERK induced by IL-6 classic signaling^36^. In addition, our model predicts that IL-6 trans-signaling induces stronger responses and additional sIL-6R shifts the signaling towards trans-signaling and promotes inflammatory responses. It is consistent with other experimental work^44,57^ and modeling work^52^ that showed greater inflammatory response induced by IL-6 trans-signaling compared to classic signaling. Specifically, Reeh et al. showed greater STAT3 activation induced by IL-6 trans-signaling than classic signaling in HepG2 cells^52^. In addition, Zegeye et al.^36^ and Lindkvist et al.^57^ showed that IL-6 trans-signaling induces greater STAT3, Akt, and ERK phosphorylation compared to classic signaling in HUVECs. Importantly, our molecularly detailed model examined this phenomenon mechanistically, which could be hard to differentiate experimentally, and found that IL-6 classic and trans-signaling actually have similar potency in inducing downstream signaling, pSTAT3, pAkt, and pERK; however, the in vitro experimental condition of abundant sIL-6R leads to stronger activation in STAT3, Akt, and ERK induced by IL-6 trans-signaling. Furthermore, our model identified the influential species and kinetic parameters that specifically modulate downstream inflammatory and/or angiogenic signals, pSTAT3, pAkt, and pERK responses, which could assist relevant experimental design to investigate the effects of potential targets.

Angiogenesis and inflammation play an important role in many diseases, such as cancer, ocular, and cardiovascular diseases. Angiogenesis also triggers inflammatory responses^17^, which leads to malfunction of endothelial cells. Specifically, endothelial cells in response to pro-inflammatory cytokines, such as IL-6, get activated resulting in increased vascular leakage and leukocyte recruitment^19,20^. However, there is a limited quantitative analysis of inflammatory pathways together with angiogenic responses in endothelial cells to inform potential treatments that target inflammation and angiogenesis. There are a number of computational models that study IL-6-induced signaling in many other cell types including hepatoma cells^52^, cardiac fibroblasts^62,63^, macrophages^64,65^, and cancer stem cells^66^. However, there is a limited quantitative understanding of IL-6 signaling in endothelial cells. Our research is the first computational model that focuses on IL-6 mediated signaling in endothelial cells to examine endothelial cytokine-mediated inflammatory and angiogenic responses.

Also, there are models that study cellular responses without considering intracellular signaling. For example, Nazari et al. linked cellular responses, specifically the temporal changes in the cancer stem cells, progenitor cells, and terminally differentiated cells with the fractional occupancy of bound receptors per cell^66^. Our model can be utilized in combination with these types of models to more accurately predict cellular behaviors as more downstream signaling species could be better indicators for cellular responses.

This model can be beneficial to study the efficiency of angiogenesis- and inflammation-based therapies. Our model can identify the important variables to the pSTAT3, pAkt and pERK levels induced by IL-6 signaling and predict how pSTAT3, pAkt, and pERK levels change by varying those parameters, which can provide quantitative insights into investigating the efficiency of targeting particular variables as angiogenesis- and inflammation-based strategies.

We do acknowledge certain limitations in our model. We adapted Reeh et al.’s IL-6 induced STAT3 pathway model which assumed that the ligand-receptor complex (Rcomplex) formed from classic and trans-signaling are the same. It implied a regulation of IL-6R and sIL-6R since the Rcomplex can associate and form both types of the receptor. Because the majority of the sIL-6R is generated by the shedding of the membrane bound IL-6R, and IL-6R expression can be regulated by ligands such as IL-6 in many cell types^67,68^, we applied Reeh et al.’s model structure to include the potential regulation of the IL-6R and sIL-6R. Additionally, we simplified many species and reactions before activating STAT3, MEK/ERK, and PI3K/Akt pathways by the stimulation of IL-6 because our main focus is their interactions. Also, we excluded soluble gp130 (sgp130) although their binding with IL-6:sIL-6R plays a role in inflammation^69^. Their contributions to the model can be incorporated in future studies. In addition, due to the scarcity of quantitative data on kinetics rates and initial conditions of IL-6 induced STAT3, Akt, and ERK activation in endothelial cells, we used parameters that govern IL-6 induced STAT3 pathway in human hepatoma cells^52^ and VEGF- and FGF-induced Akt and ERK pathways in endothelial cells^70^ as our initial guess to tune the parameters for IL-6 induced endothelial signaling, although the model was calibrated and validated using HUVEC data^44^. Moreover, we assumed that IL-6 and sIL-6 levels are constant over four hours as the nutrients in the cell culture media were still sufficient. The same assumption has been made by Reeh et al. in their IL-6 signaling model to study human hepatoma cells^52^. Also, we estimated the receptor number since the expression of IL-6R in human endothelial cells is uncertain^71^. Furthermore, as the model was calibrated to fold change experimental data, the model can predict the relative change with small variations (Fig. 2). However, it gives large variations when predicting absolute values (Figs. 3, 4). It can be improved when additional data on the receptor expression become available.

In conclusion, we developed a computational model to characterize the pSTAT3, pERK, and pAkt dynamics by the stimulation of IL-6 in endothelial cells. The model quantitatively studies STAT3, ERK, and Akt phosphorylation in response to IL-6 and sIL-6R and provides mechanical insight into inflammatory and angiogenic signaling in endothelial cells. The understanding of the regulation of inflammatory and angiogenic signals on a molecular scale can better aid the development of inflammation- and angiogenesis-based strategies.

## Methods

### Model construction

We constructed a molecular-detailed biochemical reaction network including IL-6 and their membrane-bound and soluble receptors, IL-6R and sIL-6R, respectively (Fig. 1). Signaling is induced by the IL-6 binding to their receptors and gp130, culminating with phosphorylation of STAT3, Akt, and ERK through the STAT3, PI3K/Akt, and MAPK pathways. The molecular interactions involved in the network are illustrated in Fig. 1. We adapted the IL-6 induced STAT3 pathway from the model developed by Reeh et al.^52^, and we expanded the model by including PI3K/Akt and MAPK pathways from Song and Finley’s model^70^. It is noteworthy that although STAT3 has been shown to have two phosphorylation sites, Tyr705 and Ser727^72^, it has been shown that IL-6 induced tyrosine phosphorylation depends on JAKs, while the mechanism of serine phosphorylation is not clear^73^. Thus, we only considered the singly phosphorylated STAT3 (pSTAT3) in our model. In addition, we consider that activated Akt and ERK include both singly and doubly phosphorylated forms of each species since they have been reported to get activated at two phosphorylation sites^58,74^. The model can be improved when more data are available. For simplicity, we collectively refer to these species as phosphorylated STAT3, Akt, and ERK (pSTAT3, pAkt, and pERK), respectively. The model reactions, initial conditions, and parameter values are provided in Supplementary Tables 1–3.

The network is implemented as an ordinary differential equation (ODE) model using MATLAB (MathWorks, Natick, MA). The main model includes 55 reactions, 65 species, and 68 parameters. The initial variable settings of the initial conditions and parameters involved in IL-6 induced STAT3 pathway, and the variables involved in IL-6 induced Akt and ERK pathways are taken from the median values from Reeh et al.’s calibrated model^52^ and Song and Finley’s fitted model^70^, respectively. The reactions, initial conditions, and parameter values are listed in Supplementary Tables 1 to 3. We listed four representative reactions below that describe the ligand-receptor binding as an example.

$${\rm{IL}}6+{\rm{IL}}6{\rm{R}}\mathop{\longleftrightarrow}\limits^{{\rm{p}}1{\rm{cl}},{\rm{p}}2{\rm{cl}}}\,{\rm{IL}}6:{\rm{IL}}6{\rm{R}}$$

$${\rm{IL}}6:{\rm{IL}}6{\rm{R}}+{\rm{IL}}6:{\rm{IL}}6{\rm{R}}+{\rm{gp}}130+{\rm{gp}}130\mathop{\longleftrightarrow}\limits^{{\rm{p}}3{\rm{cl}},{\rm{p}}4{\rm{cl}}}{\rm{Rcomplex}}$$

$${\rm{IL}}6+{\rm{sIL}}6{\rm{R}}\mathop{\longleftrightarrow}\limits^{{\rm{p}}1{\rm{tr}},{\rm{p}}2{\rm{tr}}}{\rm{IL}}6:{\rm{sIL}}6{\rm{R}}$$

$${\rm{IL}}6:{\rm{sIL}}6{\rm{R}}+{\rm{IL}}6:{\rm{sIL}}6{\rm{R}}+{\rm{gp}}130+{\rm{gp}}130\mathop{\longleftrightarrow}\limits^{{\rm{p}}3{\rm{tr}},{\rm{p}}4{\rm{tr}}}{\rm{Rcomplex}}$$

Because the simulated time is within four hours, we do not consider the degradation of the ligands or signaling species. The complete model is available in Supplementary File.

To set the initial conditions, since the expression of IL-6R in human endothelial cells is unclear^71^, we correlated the IL-6R level with the gp130 level, which were measured in human umbilical vein endothelial cell (HUVEC) lysates^44^ by one factor: ratio4 (*gp130*/*IL-6R* = 0.04 nM /0.0015 nM = 26) (Supplementary Table 2). Also, we assumed a negligible basal sIL-6R in the system since the basal sIL-6R level (0.00019 nM) measured in HUVEC medium is much lower than IL-6R and gp130 measured in the HUVEC lysates^44^, specifically the basal sIL-6R level is approximately 7.9-fold lower than IL-6R (0.0015 nM) and 210-fold lower than gp130 (0.04 nM).

### Sensitivity analysis

To identify the parameters and initial concentrations that significantly influence the model outputs, we performed the sensitivity analysis to calculate the Partial Rank Correlation Coefficients (PRCCs), which indicate the correlation between the model inputs and model outputs^75^. All targeted parameters and initial values were sampled simultaneously within specified bounds using Latin Hypercube Sampling (LHS); PRCC values for all targeted parameters and initial values were computed to evaluate the correlation between the model inputs (kinetic parameters or initial conditions) and the pSTAT3, pAkt, and pERK concentrations. In addition, the *p*-values from a t-distribution test corrected with Bonferroni correction were calculated. The PRCC values of the sensitive variables that are statistically significant (*p*-value < 0.05) were compared. The PRCC values can range from −1 to 1, where a higher positive PRCC value and a lower negative PRCC value indicate the input is more positively and negatively correlated to the output, respectively.

Before model training, we first calculated PRCC values for all the parameters and initial values. Since the parameters for STAT3 activation were adapted from Reeh et al.’s model^52^, these variables were sampled using LHS within the estimated lower and upper bounds from Reeh et al.’s calibrated model^52^ listed in Supplementary Table 3. All remaining model parameters and initial values were sampled within two orders of magnitude above and two orders of magnitude below the baseline values, where the baseline values were taken from the median values estimated from published literature^70,76^ listed in Supplementary Tables 2, 3. Based on the experimental data that were used for model training, we calculated the PRCC values for all the same concentrations and time points as those used in the experiments. The highest PRCC value (*PRCC*_*max*_) across all of the concentrations and time points was selected to represent the sensitivity index for each variable.

We also performed sensitivity analysis for the calibrated and validated model to identify potential targets for inflammation- and angiogenesis-based strategies.

### Identifiability analysis

In addition to parameter sensitivity, we also performed structural parameter identifiability analysis^77,78^ to consider the uncertainty caused by the model structure in a dynamical system to study molecular signal transduction. The identifiability analysis identifies the parameters that have one unique model output for each parameter value. In this method, pair-wise correlation coefficients between parameters were calculated. The identifiable parameters have correlations with all other parameters between −0.9 and 0.9 while unidentifiable parameters have correlations of >0.9 or <−0.9 with at least one other parameter.

### Data extraction

In vitro experimental data from previously published work^44,57^ that studied pSTAT3, pAkt, and pERK time course and/or dose response upon the stimulation of IL-6 with/without sIL-6R in HUVECs were selected and were used for parameter fitting and model validation. Experimental data from plots were extracted using the data extraction function *grabit*. The western blot data were extracted using ImageJ software based on the density of the protein bands.

### Parameterization

A total of 35 influential variables with *PRCC*_*max*_ values greater than 0.4 and less than −0.4 were identified by sensitivity analysis. Of these, 28 identifiable variables were identified by identifiability analysis (Supplementary Table 4, highlighted in red) Thus, we held the rest of the variables constant and estimated a total of 28 influential and identifiable variable values by fitting the model to experimental measurements^52^ using Particle Swarm Optimization (PSO) implemented by Iadevaia et al.^56^ We used MATLAB to implement the PSO algorithm. PSO starts with a population of initial particles (parameter sets). As the particles move around (i.e., as the algorithm explores the parameter space), an objective function is evaluated at each particle location. Particles communicate with one another to determine which has the lowest objective function value. The objective function for each parameter set was used to identify optimal parameter values. Specifically, we used PSO to minimize the weighted sum of squared residuals (WSSR):

1 $${\rm{WSSR}}({\rm{\theta }})=\min \mathop{\sum }\limits_{i=1}^{n}{\left(\frac{{V}_{{pred},i}({\rm{\theta }})-{V}_{\exp ,i}}{{V}_{\exp ,i}}\right)}^{2}$$

where V_*exp,i*_ is the *i*th experimental measurement, V_*pred,i*_ is the *i*th predicted value at the corresponding time point, and *n* is the total number of experimental data points. The minimization is subject to ${\rm{\theta }}$, the set of upper and lower bounds on each of the fitted parameters. The bounds for the parameters involved in the reactions for STAT3 activation were set to be the estimated lower and upper bounds from Reeh et al.’s calibrated model^52^ and listed in Supplementary Table 3. Also since the dissociation constant (Kd) of IL-6 for IL-6R has been reported to be 0.5–50 nM^52^, we set the upper and lower bounds on p2cl to be 0.5*p1cl and 50*p1cl to confine the Kd for reaction IL-6 + IL-6R $\longleftrightarrow$ IL-6:IL-6R. In addition, the bounds for the remaining model parameters and initial values were set to be two orders of magnitude above and below the baseline parameter values, which were taken from the median values estimated from literature^70,76^ and listed in Supplementary Tables 2–3.

The model was fitted using five experimental datasets from the literature^44^, specifically: (1) relative change of pSTAT3 time course response from 0 to 240 min stimulated by 50 ng/ml IL-6 alone and in combination with 100 ng/ml sIL-6R compared with a reference point (pSTAT3 stimulated by 50 ng/ml IL-6 in combination with 100 ng/ml sIL-6R at 5 min); (2) relative change of ppAkt time course response from 0 to 240 min stimulated by 50 ng/ml IL-6 alone and in combination with 100 ng/ml sIL-6R compared with a reference point (ppAkt stimulated by 50 ng/ml IL-6 in combination with 100 ng/ml sIL-6R at 5 min); (3) relative change of pERK time course response from 0 to 240 min stimulated 50 ng/ml IL-6 alone and in combination with 100 ng/ml sIL-6R compared with a reference time point (pERK stimulated by 50 ng/ml IL-6 in combination with 100 ng/ml sIL-6R at 5 min); (4) relative change of pSTAT3 dose response stimulated by varying concentrations IL-6 from 0 to 50 ng/ml at 15 min compared with a reference point (pSTAT3 stimulated by 10 ng/ml IL-6 alone at 15 min); (5) relative change of pSTAT3 dose response stimulated by varying concentrations IL-6 from 0 to 50 ng/ml in combination with 100 ng/ml sIL-6R at 15 min compared with a reference point (pSTAT3 stimulated by 50 ng/ml IL-6 alone at 15 min). All published experiments were conducted using human umbilical vein endothelial cells (HUVECs)^44^.

Model simulations were compared to experimental measurements. Specifically, the relative change of the responses was calculated as following:

2 $${\rm{relative}}\,{\rm{change}}({\rm{t}}{,c}_{{IL}6}{,c}_{{sIL}6R})=\frac{{response}({\rm{t}}{,c}_{{IL}6}{,c}_{{sIL}6R})-{{response}}(t_{{ref}}{,c}_{{ref}\_{IL}6}{,c}_{{ref}\_{sIL}6R})}{{{response}}(t_{{ref}}{,c}_{{ref}\_{IL}6}{,c}_{{ref}\_{sIL}6R})}$$

where ${response}\left({\rm{t}}{,c}_{{IL}6}{,c}_{{sIL}6R}\right)$ is the level of pSTAT3, ppAkt, or pERK upon the stimulation of concentration ${c}_{{IL}6}$ IL-6 in combination of concentration ${c}_{{sIL}6R}$ sIL-6R at time t, and ${{response}(t}_{{ref}}{,c}_{{ref\_IL}6}{,c}_{{ref\_sIL}6R})$ is the response (pSTAT3, ppAkt, or pERK) upon the stimulation of a reference concentration combination of ${c}_{{ref\_IL}6}$ IL-6 and ${c}_{{ref\_sIL}6R}$ sIL-6R at a reference time point *t*_*ref*_.

Here, the pSTAT3 in the model simulation includes all free and bound forms of singly- phosphorylated STAT3. Also, ppAkt includes all free and bound forms of doubly-phosphorylated Akt, since Zegeye et al. and Lindkvist et al. used anti-phospho-AKT^Ser473^ antibody for detecting phosphorylated Akt^44,57^ and it has been reported that Akt gets phosphorylated at S473 as a secondary event^60,79,80^. Thus, we compared the predicted doubly phosphorylated Akt (ppAkt) to experimental data^44,57^. In addition, pERK in the model simulation includes all free and bound forms of singly- and doubly- phosphorylated ERK.

#### Constraints

In order to capture the whole dynamics of pSTAT3, pAkt, and pERK within 240 min, we applied a constraint for the relative change for ppAkt and pERK induced by IL-6 trans-signaling at 120 and 240 min by a factor of 0.01 when calculating the WSSR. Since the experimental relative change for ppAkt (0.32, and −0.0087) and pERK (0.14, and −0.26) induced by IL-6 trans-signaling at later time points are relatively low compared to other time points as they are reaching a plateau level after 100 min, we reduced their WSSR by a factor of 0.01 to let the model be more able to capture the whole dynamics rather than only the plateau behavior. Also, we increased the WSSR for pSTAT3 induced by IL-6 stimulation alone at 60, 120, and 240 min and trans-signaling at 120 and 240 min by a factor of 100 to better capture the whole dynamics of pSTAT3.

In addition, compared to a total of 48 time course data points, there are only 14 dose response data points. To better capture the dose response, we added a weight of 400 and 100 when calculating the WSSR for pSTAT3 dose response induced by IL-6 stimulation alone and trans-signaling at 0, 0.1, 1, 2, 5, and 10 ng/ml.

We first fitted the model 100 times to the experimental data. However, from the parameter set that has the lowest errors, many fitted values were found at one of the bounds (Supplementary Table 5). To exclude the possibility of arbitrary bounds limiting the parameter search space, we adjusted the bounds to be two orders of magnitude above and below the set of parameter values that has the lowest error (Supplementary Table 6). The identified influential variables were estimated another 600 times with the new bounds. With the second round of fitting, none of the parameters were estimated to be at one of the bounds (Supplementary Table 6). After model training, we validated the model with three datasets not used in the fitting. We predicted the 10 ng/ml IL-6 alone and in combination with 10 ng/ml sIL-6R induced pSTAT3, ppAkt, and pERK relative change time course responses using the reference points, pSTAT3, ppAkt, and pERK stimulated by 10 ng/ml IL-6 alone and in combination of 10 ng/ml sIL-6R at 10 min, respectively^57^. The experiments^57^ used for validation were performed using HUVECs.

#### Goodness of fit

The performance of the model was assessed as WSSR between the model predictions and experimental data and a run test was to determine if the predicted curve deviates systematically from the experimental data^81,82^.

For all three datasets used for validation, we simulated the experimental conditions without any additional model fitting and compared to the experimental measurements. A total of 12 parameter sets with the smallest errors and *p*-values greater than 0.05 by performing the runs test were taken to be the “best” sets based on the model fitting and validation (Supplementary Table 6) and were used for all model simulations.

If a fit is appropriate for the experimental measurements, the residuals only represent experimental error, which would have a random arrangement of positive and negative residuals; whereas the residuals that have the same signs would tend to cluster if the fit is inappropriate^81,82^. A run test determines whether the data are systematically different from the predictions^81,82^. A *p*-value lower than 0.05 indicates the predicted curve deviates systematically from the experimental data, while a *p*-value greater than 0.05 suggests the residuals appear randomly distributed across the zero line^81,82^.

### Monte Carlo simulations

To study the robustness of the system, the fitted model was run 1000 times by generating 1000 values for all parameters and non-zero initial concentrations, sampling from normal and lognormal distributions, respectively. For initial concentrations and parameters that were estimated by fitting to the experimental data, the mean values (μ) were the best fit, and for all other model variable values, we set μ to be the baseline values. The variances for the initial concentrations were set as an estimate of 10%μ. For all the parameters, we calculated the standard deviation (σ) to capture 99.7% of the possible values given the range of μ ± 50%μ (i.e., μ ± 3σ). It is worth noting that with this sampling, it is possible to get negative values, though this is unlikely to occur. However, if any negative values were selected, we resampled until all the sampled variables were positive.

### Signaling responses

We investigated the STAT3, Akt, and ERK phosphorylation responses upon stimulation by IL-6 classic- and/or trans-signaling.

#### Maximum pSTAT3, pAkt, and pERK

We calculated the maximum STAT3, Akt, and ERK phosphorylation levels induced by the stimulation of IL-6 classic- and/or trans-signaling within four hours.

#### Area under the curve (AUC) of pSTAT3, pAkt, and pERK

We calculated the AUC of STAT3, Akt, and ERK phosphorylation levels induced by the stimulation by IL-6 classic- and/or trans-signaling within four hours.

#### Reaction rates

We specified the rates of each reaction based on the law of mass action, where the rate of a chemical reaction is proportional to the amount of each reactant. For example, for the binding of IL-6 to IL-6R:

$${\rm{IL}}6+{\rm{IL}}6{\rm{R}}\mathop{\longleftrightarrow}\limits^{{\rm{p}}1{\rm{cl}},{\rm{p}}2{\rm{cl}}}{\rm{IL}}6:{\rm{IL}}6{\rm{R}}$$

The reaction rate is:

3 $${\rm{Rate}}={\rm{p}}1{\rm{cl}}{\cdot} {IL}6{\cdot} {IL}6R-{\rm{p}}2{\rm{cl}}{\cdot} {IL}6:{IL}6R$$

Here $p1{cl}$ and $p2{cl}$ are rate constants for the forward and reverse reactions, respectively, and *IL6*, *IL6R*, and *IL6:IL6R* are the species’ concentrations.

### Supplementary information

Supplementary File

Supplementary Tables

nr-reporting-summary

## Data Availability

All data generated or analyzed during this study are included in this published article and its supplementary materials.

## Code availability

The model has been constructed using MATLAB (version R2021b). All model reactions, initial conditions, and values of parameters are provided in Supplementary Tables 1 to 3 in Supplementary Data. MATLAB.m file containing model code is available in the Supplementary File.
