# Supplementary material for: Experiment-based computational model predicts that IL-6 classic and trans-signaling exhibit similar potency in inducing downstream signaling in endothelial cells
Source: NPJ Syst Biol Appl. 2023 Sep 21;9:45. doi: 10.1038/s41540-023-00308-2 (PMC10514195; doi:10.1038/s41540-023-00308-2)
Supplement: Supplementary file 1 — Supplementary File [file 41540_2023_308_MOESM1_ESM.pdf]

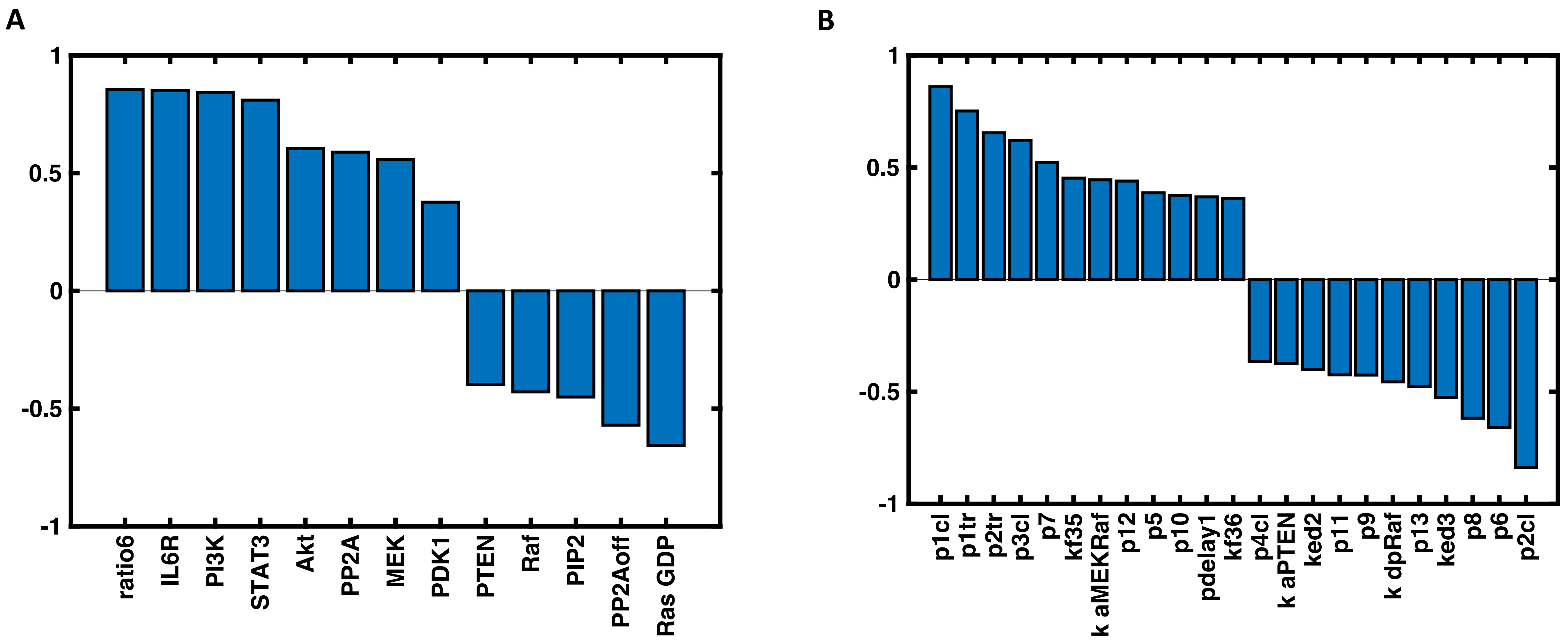

Supplementary Figure 1. Results of sensitivity analysis for model training. PRCC values for influential initial concentrations (A) and parameters (B). Y axes indicate PRCC values.

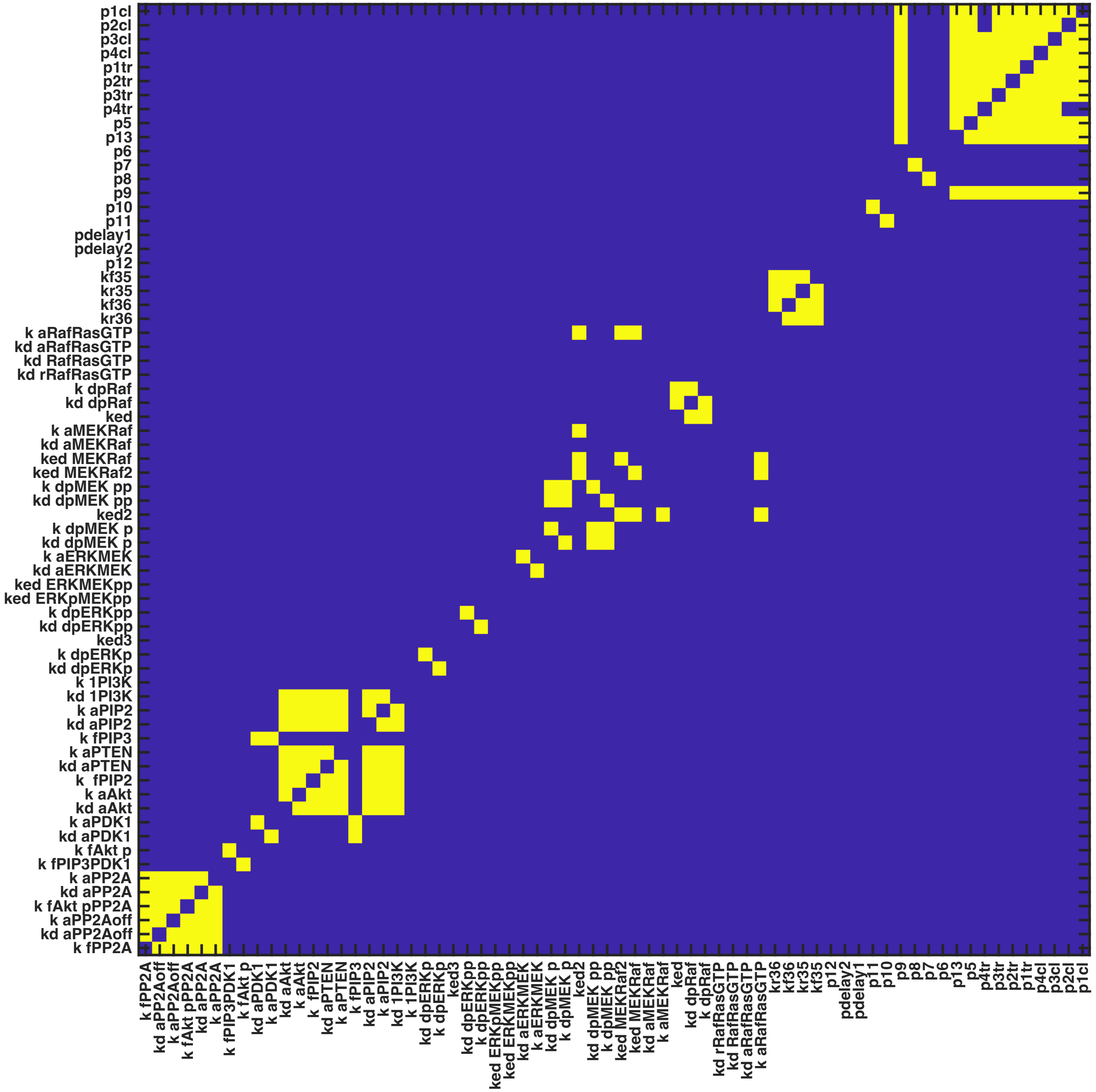

Supplementary Figure 2. Results of identifiability analysis. identifiability analysis identifies the parameters that have one unique model output for each parameter value. In this method, pair-wise correlation coefficients between parameters were calculated. The identifiable parameters having correlations with all other parameters between -0.9 and 0.9 are labeled blue. The unidentifiable parameters having correlations of > 0.9 or < -0.9 with at least one other parameter are labeled yellow.

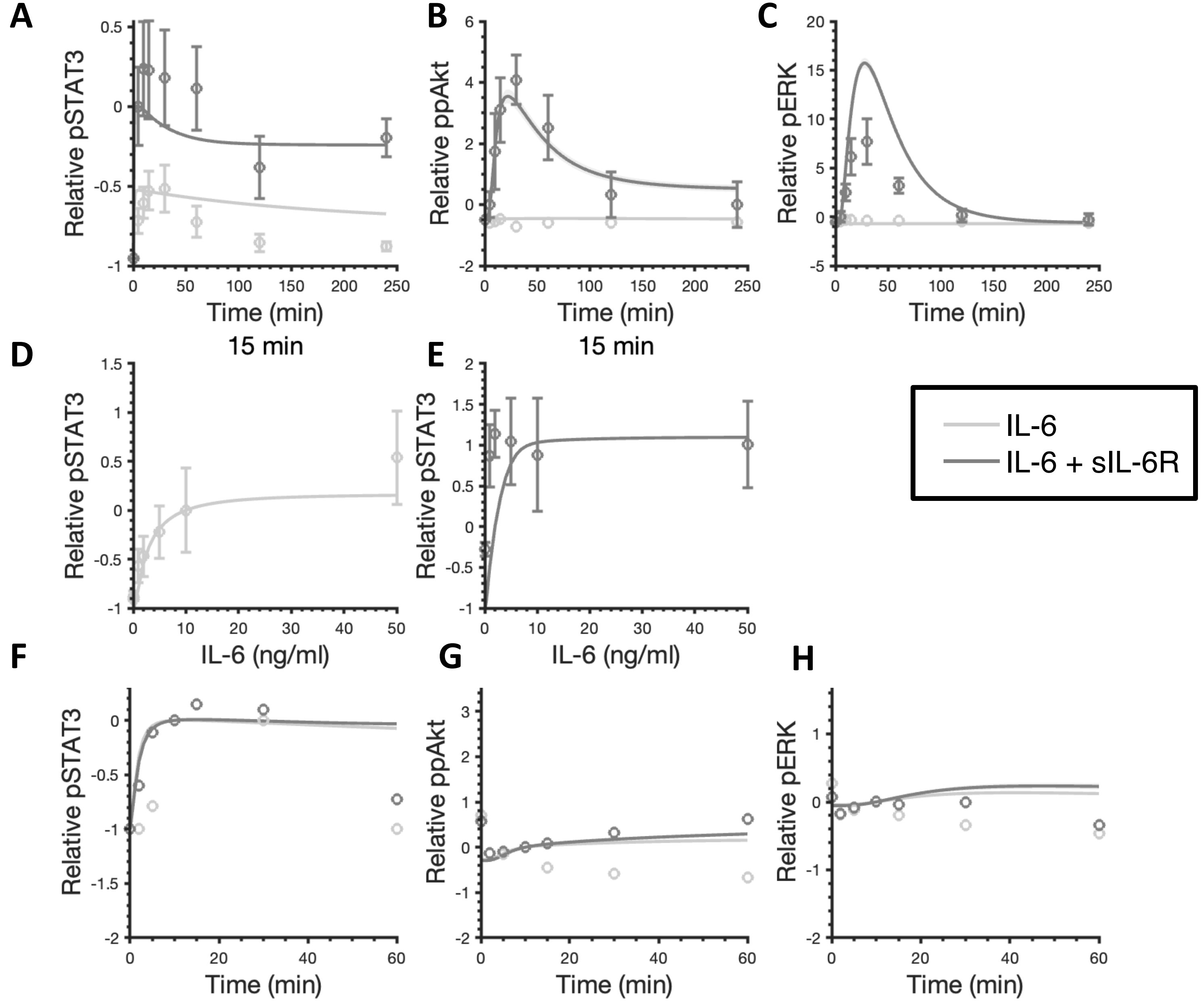

Supplementary Figure 3. Monte Carlo simulations for training and validation data for IL-6 alone and in combination with sIL-6R stimulation. 50 ng/ml IL-6 with or without additional 100 ng/ml sIL-6R induced relative pSTAT3 (A), ppAkt (B), and pERK (C). Varying concentrations of IL-6 alone induced relative pSTAT3 (D) and with additional 100 ng/ml sIL-6R induced relative pSTAT3 (E). The circles are experimental data. Bars are mean ± SEM. Curves are the mean values of the 1,000 Monte Carlo simulations. Shaded regions show 95% confidence intervals. Light gray: 50 ng/ml IL-6 (A-C), 0 – 50 ng/ml IL-6 (D), and 10 ng/ml IL-6 (F-H) stimulation; Dark gray: 50 ng/ml IL-6 + 100 ng/ml sIL-6R (A-C), 0 – 50 ng/ml IL-6 + 100 ng/ml sIL-6R (E), and 10 ng/ml IL-6 + 10 ng/ml sIL-6R (F-H) stimulation.

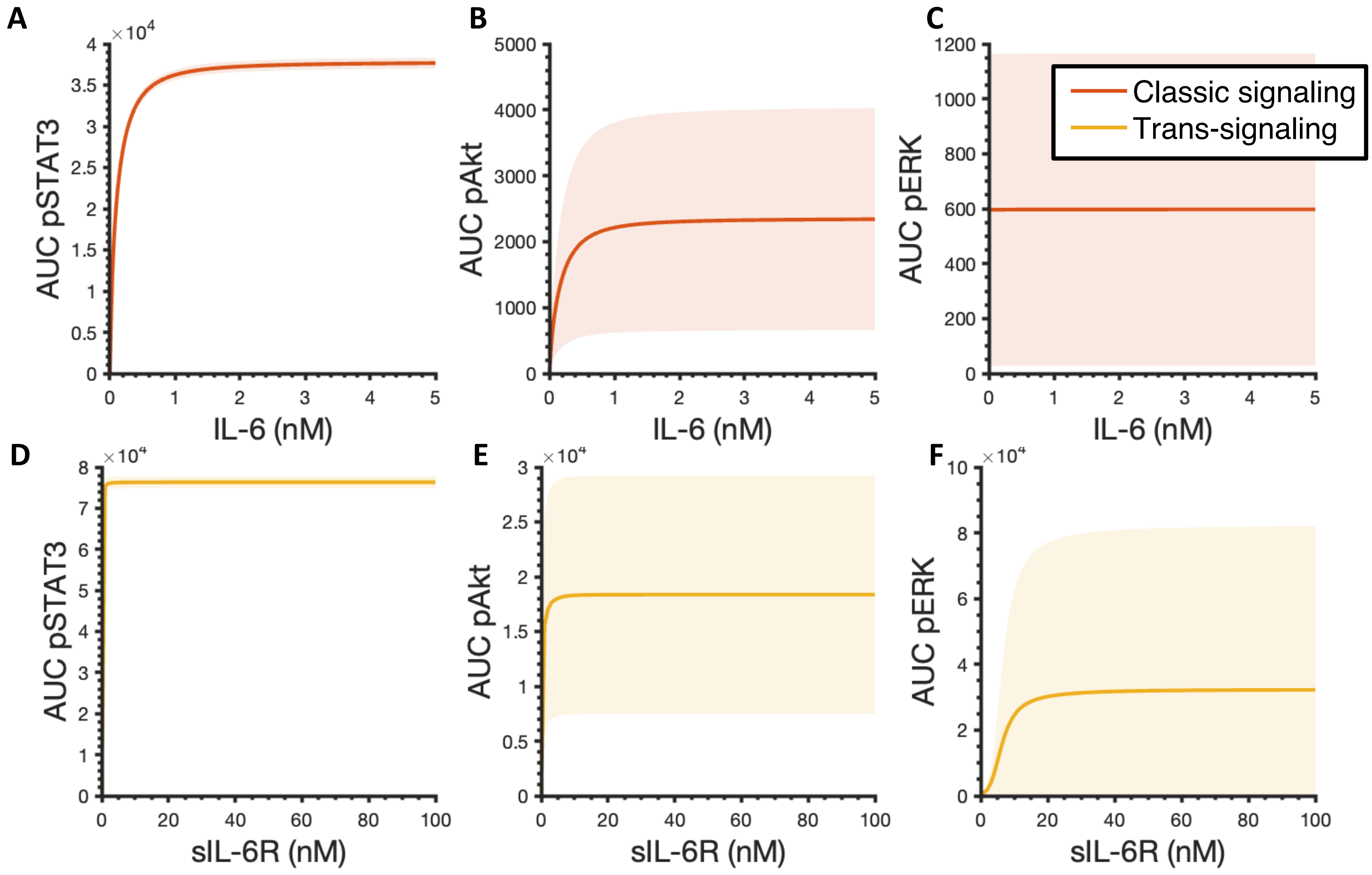

Supplementary Figure 4. Predicted AUC of pSTAT3, pAkt, and pERK responses. AUC of pSTAT3 (A), pAkt (B), and pERK (C) in response to IL-6 concentrations varying from 0 to 5 nM without sIL-6R. In the absence of IL-6R, 0.2 nM IL-6 in combination with sIL-6R concentrations varying from 0 to 100 nM induced AUC of pSTAT3 (D), pAkt (E), and pERK (F). Curves are the mean values of the 12 best fits. Shaded regions show 95% confidence intervals of the fits. Orange: classic signaling responses; Yellow: trans-signaling responses.

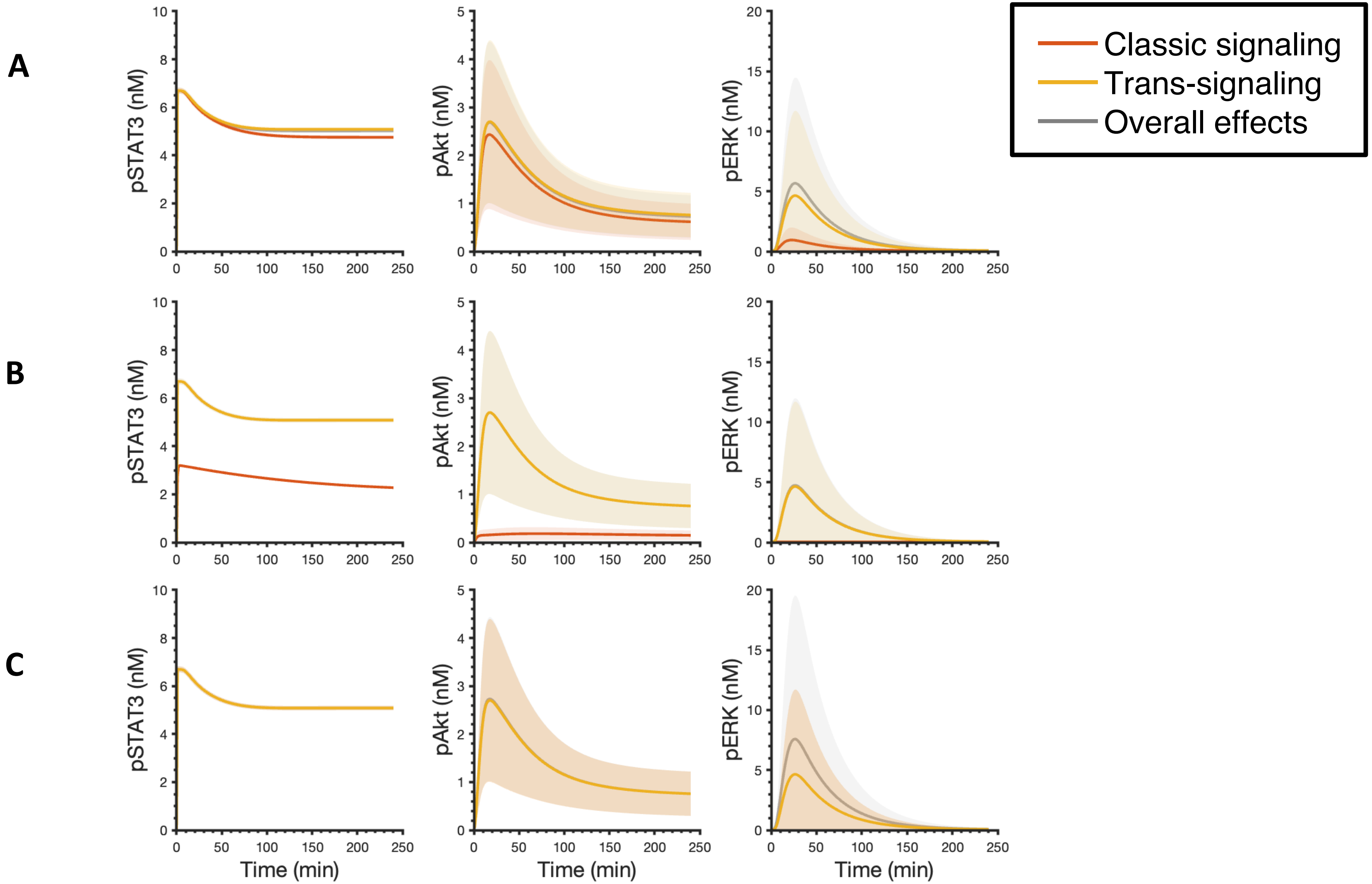

Supplementary Figure 5. Predicted time courses of pSTAT3, pAkt, and pERK following stimulation by 2 nM IL-6 alone with a mean value of 6.4 nM IL-6R (orange), 2 nM IL-6 in combination with a mean value of 6.4 nM sIL-6R in the absence of IL-6R (yellow), and 2 nM IL-6 with a mean value of 6.4 nM of both IL-6R and sIL-6R (gray) when IL-6R and sIL-6R are set at the same level and remain constant within four-hour simulation time (A), when kinetic rates governing R1 and R2 to be the same as the corresponding kinetic rates for R3 and R4 (B), and when both IL-6R was set as a constant input and kinetic rates governing R1 and R2 to be the same as the corresponding kinetic rates for R3 and R4 (C). Curves are the mean values of the 12 best fits. Shaded regions show 95% confidence intervals of the fits. Orange: classic signaling responses; Yellow: trans-signaling responses; Gray: overall responses. R1: IL-6 + IL-6R ↔ IL-6:IL-6R; R2: 2 IL-6:IL-6R + 2 gp130 ↔ Rcomplex; R3: IL-6 + sIL-6R ↔ IL-6:sIL-6R; R4: 2 IL-6:sIL-6R + 2 gp130 ↔

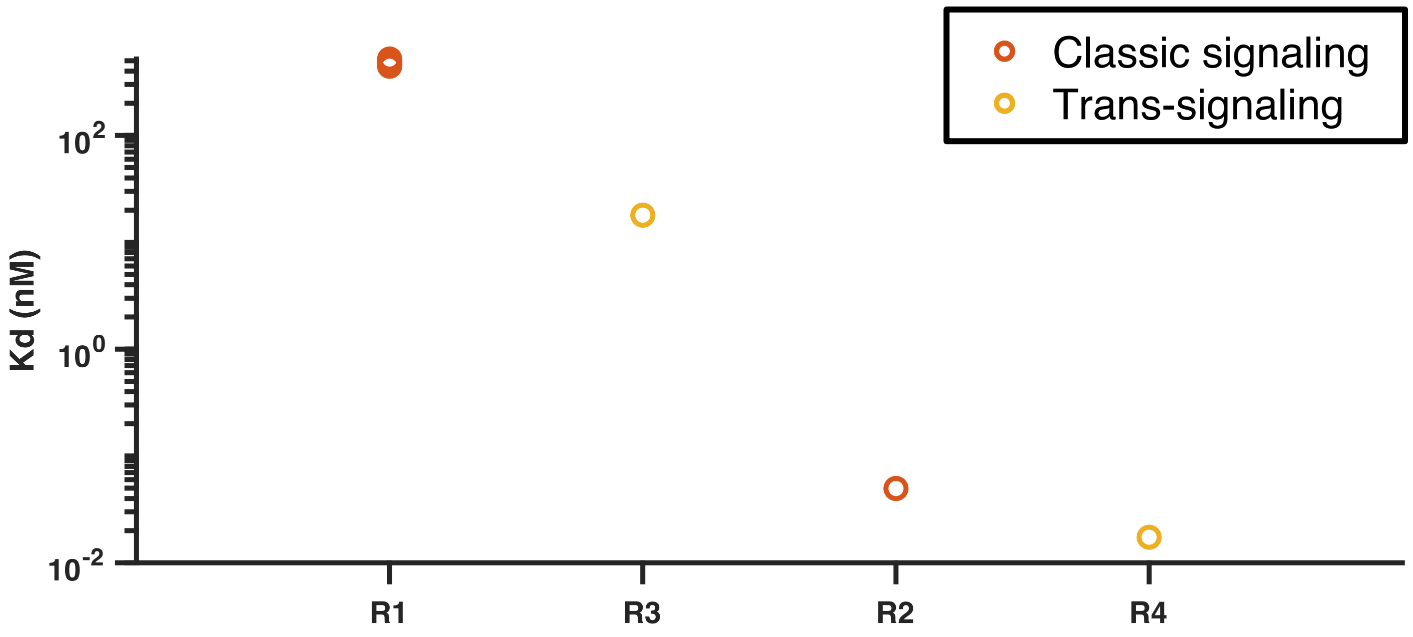

Supplementary Figure 6. Dissociation constants (Kd) of ligand-receptor binding. R1: IL-6 + IL-6R ⟷ IL-6:IL-6R; R2: 2 IL-6:IL-6R + 2 gp130 ⟷ Rcomplex; R3: IL-6 + sIL-6R ⟷ IL-6:sIL-6R; R4: 2 IL-6:sIL-6R + 2 gp130 ⟷ Rcomplex. Each circle represents one fit. Orange: classic signaling responses; Yellow: trans-signaling responses.

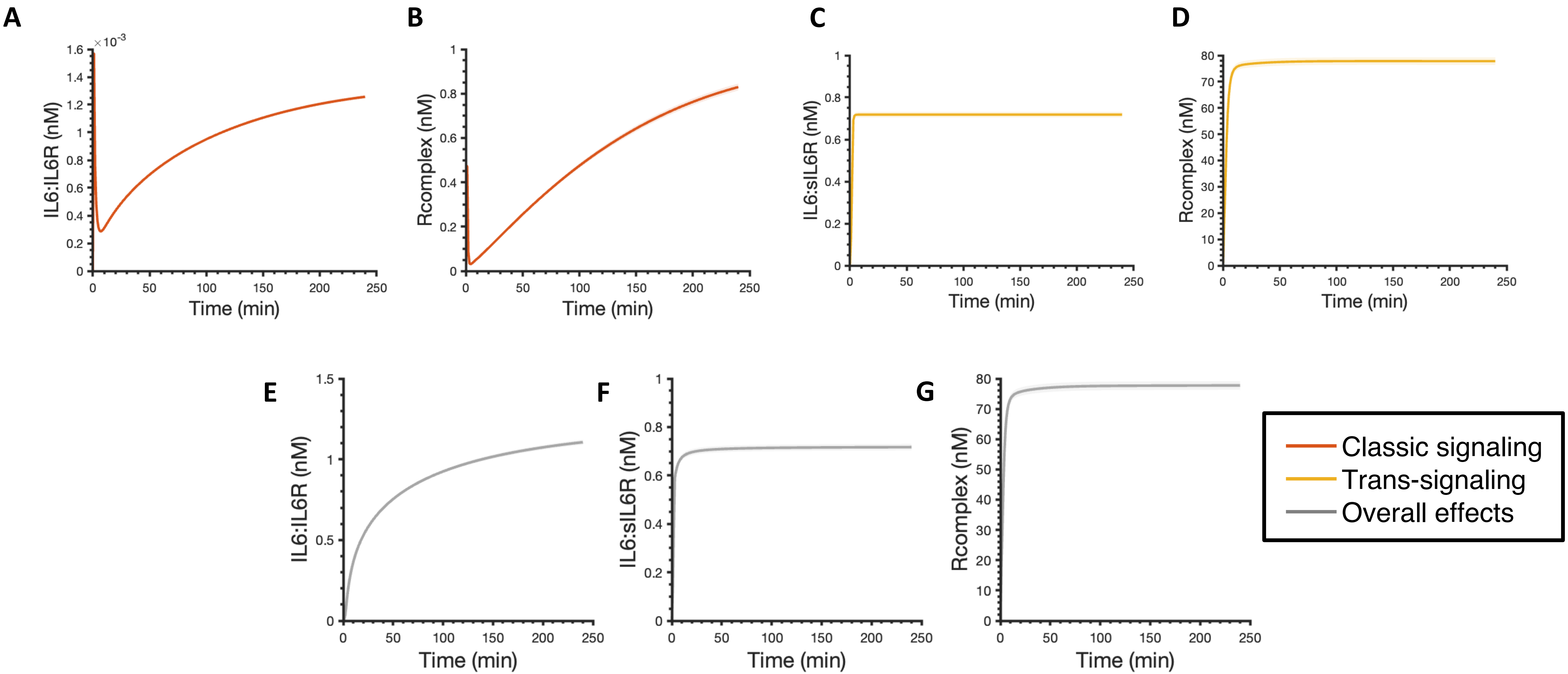

Supplementary Figure 7. Dynamics of relevant species involved in ligand-receptor binding following stimulation by 2 nM IL-6 alone with a mean value of 6.4 nM IL-6R (orange) (A-B), 2 nM IL-6 in combination with a mean value of 6.4 nM sIL-6R in the absence of IL-6R (yellow) (C-D), and 2 nM IL-6 with a mean value of 6.4 nM of both IL-6R and sIL-6R (gray) (E-G). Curves are the mean values of the 12 best fits. Shaded regions show 95% confidence intervals of the fits.

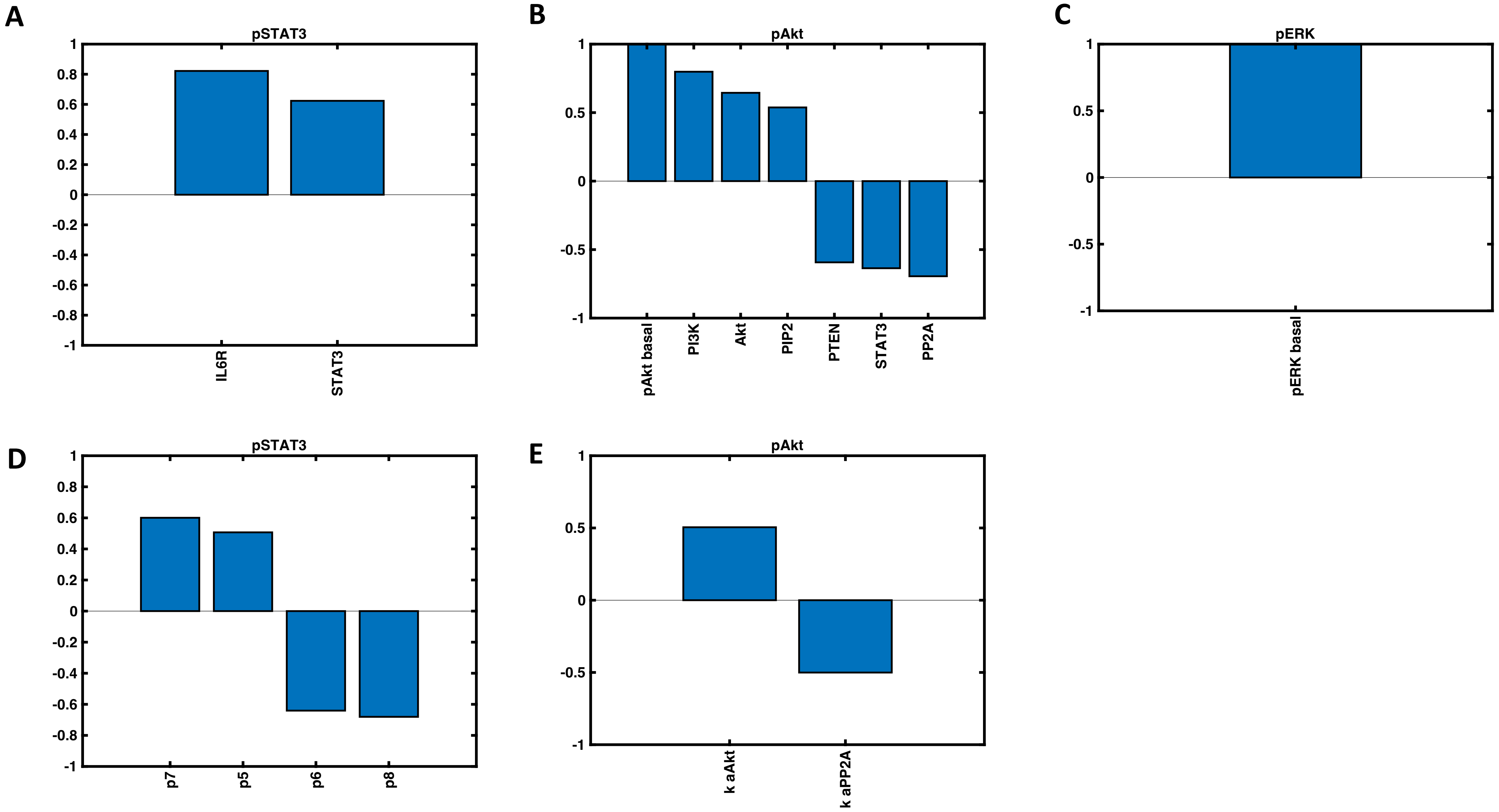

Supplementary Figure 8. Results of sensitivity analysis for the trained and validated model. PRCC values that are greater than 0.5 or less than -0.5 for influential initial concentrations pSTAT3 (A), pAkt (B), and pERK, and influential parameters to pSTAT3 (D) and pAkt (E). Y axes indicate PRCC values. Note, no parameters were identified as influential for pERK.

MATLAB.m file containing model code

```
function dsdt = coreFile_IL6(s,y,params)

IL6	=	y(	1	);
IL6R	=	y(	2	);
IL6_IL6R	=	y(	3	);
gp130	=	y(	4	);
Rcomplex =	y(	5	);
sIL6R	=	y(	6	);
IL6_sIL6R	=	y(	7	);
Rcomplex_a =	y(	8	);
SOCS3	=	y(	9	);
Rcomplex_a_Ras_GDP	=	y(	10 );
STAT3 =	y(	11	);
pSTAT3	=	y(	12	);
SOCS3mRNA_1	=	y(	13	);
SOCS3mRNA_2	=	y(	14	);
SOCS3mRNA =	y(	15	);
SOCS3_1	=	y(	16	);
SOCS3_2	=	y(	17	);
Rcomplex_a_pPI3K =	y(	18	);
Ras_GDP	=	y(	19	);
Rcomplex_a_pPI3K_PIP2 =	y(	20	);
Ras_GTP	=	y(	21	);
PI3K = y(	22	);
MEK	=	y(	23	);
ERK	=	y(	24	);
MEKpp	=	y(	25	);
ERKpp =	y(	26	);
MEKpp_ERK	=	y(	27	);
pERK	=	y(	28	);
pERK_ppMEK =	y(	29	);
pMEK	=	y(	30	);
Ptase2 =	y(	31	);
Ptase3 =	y(	32	);
```

```
ppMEK_Ptase2	=	y(	33	);
pMEK_Ptase2	=	y(	34	);
ppERK_Ptase3	=	y(	35	);
pERK_Ptase3	=	y(	36	);
aRas_GTP	=	y(	37	);
Raf	=	y(	38	);
Raf_Ras_GTP	=	y(	39	);
aRaf	=	y(	40	);
Ptase1	=	y(	41	);
aRaf_Ptase1	=	y(	42	);
MEK_aRaf	=	y(	43	);
pMEK_aRaf	=	y(	44	);
PIP3	=	y(	45	);
PTEN	=	y(	46	);
PIP3_PTEN	=	y(	47	);
Akt	=	y(	48	);
PIP3_Akt	=	y(	49	);
PDK1	=	y(	50	);
PIP3_Akt_PDK1	=	y(	51	);
pAkt	=	y(	52	);
PIP3_PDK1	=	y(	53	);
PIP3_pAkt	=	y(	54	);
PIP3_pAkt_PDK1	=	y(	55	);
ppAkt	=	y(	56	);
PP2A	=	y(	57	);
ppAkt_PP2A	=	y(	58	);
pAkt_PP2A	=	y(	59	);
PP2Aoff	=	y(	60	);
ppAkt_PP2Aoff	=	y(	61	);
PIP2	=	y(	62	);

p1cl	=	params(	1	,1);
p2cl	=	params(	2	,1);
p3cl	=	params(	3	,1);
p4cl	=	params(	4	,1);
p1tr	=	params(	5	,1);
```

```
p2tr	=	params(	6	,1);
p3tr	=	params(	7	,1);
p4tr	=	params(	8	,1);
p5	=	params(	9	,1);
p13	=	params(	10	,1);
p6	=	params(	11	,1);
p7	=	params(	12	,1);
p8	=	params(	13	,1);
p9	=	params(	14	,1);
p10	=	params(	15	,1);
p11	=	params(	16	,1);
pdelay1		=	params(	17	,1);
pdelay2		=	params(	18	,1);
p12	=	params(	19	,1);
kf35	=	params(	20	,1);
kr35	=	params(	21	,1);
kf36	=	params(	22	,1);
kr36	=	params(	23	,1);
k_aRafRasGTP		=	params(	24	,1);
kd_aRafRasGTP		=	params(	25	,1);
kd_RafRasGTP		=	params(	26	,1);
kd_rRafRasGTP		=	params(	27	,1);
k_dpRaf		=	params(	28	,1);
kd_dpRaf		=	params(	29	,1);
ked	=	params(	30	,1);
k_aMEKRaf	=	params(	31	,1);
kd_aMEKRaf	=	params(	32	,1);
ked_MEKRaf	=	params(	33	,1);
ked_MEKRaf2		=	params(	34	,1);
k_dpMEK_pp	=	params(	35	,1);
kd_dpMEK_pp		=	params(	36	,1);
ked2	=	params(	37	,1);
k_dpMEK_p	=	params(	38	,1);
kd_dpMEK_p	=	params(	39	,1);
k_aERKMEK	=	params(	40	,1);
kd_aERKMEK=	params(	41	,1);
```

```
ked_ERKMEKpp      =     params(     42     ,1);
ked_ERKpMEKpp     =     params(     43     ,1);
k_dpERKpp    =     params(     44     ,1);
kd_dpERKpp   =     params(     45     ,1);
ked3    =     params(     46     ,1);
k_dpERKp     =     params(     47     ,1);
kd_dpERKp    =     params(     48     ,1);
k_1PI3K      =     params(     49     ,1);
kd_1PI3K     =     params(     50     ,1);
k_aPIP2      =     params(     51     ,1);
kd_aPIP2     =     params(     52     ,1);
k_fPIP3      =     params(     53     ,1);
k_aPTEN      =     params(     54     ,1);
kd_aPTEN     =     params(     55     ,1);
k_fPIP2      =     params(     56     ,1);
k_aAkt  =     params(     57     ,1);
kd_aAkt      =     params(     58     ,1);
k_aPDK1      =     params(     59     ,1);
kd_aPDK1     =     params(     60     ,1);
k_fAkt_p     =     params(     61     ,1);
k_fPIP3PDK1  =     params(     62     ,1);
k_aPP2A      =     params(     63     ,1);
kd_aPP2A     =     params(     64     ,1);
k_fAkt_pPP2A      =     params(     65     ,1);
k_aPP2Aoff   =     params(     66     ,1);
kd_aPP2Aoff  =     params(     67     ,1);
k_fPP2A      =     params(     68     ,1);

R1    =     p1cl * IL6 * IL6R - p2cl * IL6_IL6R    ;
R2    =     p3cl * (IL6_IL6R)^2 * (gp130)^2 - p4cl * Rcomplex   ;
R3    =     p1tr * IL6 *sIL6R - p2tr * IL6_sIL6R   ;
R4    =     p3tr * (IL6_sIL6R)^2 * (gp130)^2 - p4tr * Rcomplex  ;
R5    =     p9 * ((pSTAT3)^2) *(1 +pSTAT3)    ;
R6    =     pdelay1 * SOCS3mRNA_1    ;
R7    =     pdelay1 * SOCS3mRNA_2    ;
R8    =     p10 * SOCS3mRNA   ;
```

R9 = p11 * SOCS3mRNA ;
R10 = pdelay2 * SOCS3_1 ;
R11 = pdelay2 * SOCS3_2 ;
R12 = p12 * SOCS3 ;
R13 = k_aRafRasGTP*Raf*Ras_GTP-kd_aRafRasGTP*Raf_Ras_GTP ;
R14 = kd_RafRasGTP*Raf_Ras_GTP-kd_rRafRasGTP*aRaf*aRas_GTP;
R15 = k_dpRaf*aRaf*Ptase1-kd_dpRaf*aRaf_Ptase1 ;
R16 = ked*aRaf_Ptase1 ;
R17 = k_aMEKRaf*MEK*aRaf-kd_aMEKRaf*MEK_aRaf ;
R18 = ked_MEKRaf*MEK_aRaf ;
R19 = k_aMEKRaf*pMEK*aRaf-kd_aMEKRaf *pMEK_aRaf ;
R20 = ked_MEKRaf2*pMEK_aRaf ;
R21 = k_dpMEK_pp*MEKpp*Ptase2-kd_dpMEK_pp*ppMEK_Ptase2;
R22 = ked2*ppMEK_Ptase2 ;
R23 = k_dpMEK_p*pMEK*Ptase2-kd_dpMEK_p*pMEK_Ptase2 ;
R24 = ked2*pMEK_Ptase2 ;
R25 = k_aERKMEK*MEKpp*ERK - kd_aERKMEK*MEKpp_ERK ;
R26 = ked_ERKMEKpp*MEKpp_ERK ;
R27 = k_aERKMEK*pERK*MEKpp - kd_aERKMEK*pERK_ppMEK ;
R28 = ked_ERKpMEKpp*pERK_ppMEK ;
R29 = k_dpERKpp*ERKpp*Ptase3-kd_dpERKpp*ppERK_Ptase3;
R30 = ked3*ppERK_Ptase3 ;
R31 = k_dpERKp*pERK*Ptase3-kd_dpERKp*pERK_Ptase3 ;
R32 = ked3*pERK_Ptase3 ;
R33 = k_aPTEN*PIP3*PTEN-kd_aPTEN*PIP3_PTEN ;
R34 = k_fPIP2*PIP3_PTEN ;
R35 = k_aAkt*PIP3*Akt-kd_aAkt*PIP3_Akt ;
R36 = k_aPDK1*PIP3_Akt*PDK1-kd_aPDK1*PIP3_Akt_PDK1 ;
R37 = k_fAkt_p*PIP3_Akt_PDK1 ;
R38 = k_fPIP3PDK1*PIP3_PDK1 ;
R39 = k_aAkt*PIP3*pAkt- kd_aAkt*PIP3_pAkt ;
R40 = k_aPDK1 *PIP3_pAkt*PDK1-kd_aPDK1 *PIP3_pAkt_PDK1;
R41 = k_fAkt_p*PIP3_pAkt_PDK1 ;
R42 = k_aPP2A *ppAkt*PP2A-kd_aPP2A *ppAkt_PP2A ;
R43 = k_fAkt_pPP2A * ppAkt_PP2A;
R44 = k_aPP2A *pAkt*PP2A-kd_aPP2A *pAkt_PP2A ;

```
R45	=	k_fAkt_pPP2A *pAkt_PP2A	;
R46	=	k_aPP2Aoff *ppAkt*PP2Aoff-kd_aPP2Aoff *ppAkt_PP2Aoff		;
R47	=	k_fPP2A *ppAkt_PP2Aoff	;
R48	=	p5 * Rcomplex/(1 +p13*SOCS3)	;
R49	=	p6*Rcomplex_a	;
R50	=	p7*Rcomplex_a *STAT3 - p8 * pSTAT3	;
R51	=	kf35* Rcomplex_a * Ras_GDP - kr35* Rcomplex_a_Ras_GDP	;
R52	=	kf36 * Rcomplex_a_Ras_GDP -  kr36 * Rcomplex_a  * Ras_GTP	;
R53	=	k_1PI3K*Rcomplex_a * PI3K - kd_1PI3K * Rcomplex_a_pPI3K	;
R54	=	k_aPIP2 * Rcomplex_a_pPI3K * PIP2 - kd_aPIP2 * Rcomplex_a_pPI3K_PIP2;
R55	=	k_fPIP3* Rcomplex_a_pPI3K_PIP2	;

dsdt(	1	,1)	=	0	;
dsdt(	2	,1)	=	-R1	;
dsdt(	3	,1)	=	R1-2*R2	;
dsdt(	4	,1)	=	-2*R2-2*R4	;
dsdt(	5	,1)	=	R2+R4-R48+R49	;
dsdt(	6	,1)	=	0	;
dsdt(	7	,1)	=	R3-2*R4	;
dsdt(	8	,1)	=	R48-R49 -R51+R52-R50 -R53	;
dsdt(	9	,1)	=	R11-R12	;
dsdt(	10	,1)	=	R51 -R52	;
dsdt(	11	,1)	=	-R50	;
dsdt(	12	,1)	=	R50	;
dsdt(	13	,1)	=	R5-R6	;
dsdt(	14	,1)	=	R6-R7	;
dsdt(	15	,1)	=	R7-R8	;
dsdt(	16	,1)	=	R9-R10	;
dsdt(	17	,1)	=	R10-R11	;
dsdt(	18	,1)	=	R53-R54+R55	;
dsdt(	19	,1)	=	-R51	;
dsdt(	20	,1)	=	R54-R55	;
dsdt(	21	,1)	=	R52-R13	;
dsdt(	22	,1)	=	-R53	;
dsdt(	23	,1)	=	-R17+R24	;
dsdt(	24	,1)	=	-R25+R32	;
```

```
dsdt(	25	,1)	=	R20-R21-R25+R26-R27+R28	;
dsdt(	26	,1)	=	R28-R29	;
dsdt(	27	,1)	=	R25-R26	;
dsdt(	28	,1)	=	R26-R27+R30-R31	;
dsdt(	29	,1)	=	R27-R28	;
dsdt(	30	,1)	=	R18-R19+R22-R23	;
dsdt(	31	,1)	=	-R21+R22-R23+R24	;
dsdt(	32	,1)	=	-R29+R30-R31+R32	;
dsdt(	33	,1)	=	R21-R22	;
dsdt(	34	,1)	=	R23-R24	;
dsdt(	35	,1)	=	R29-R30	;
dsdt(	36	,1)	=	R31-R32	;
dsdt(	37	,1)	=	R14	;
dsdt(	38	,1)	=	-R13+R16	;
dsdt(	39	,1)	=	R13-R14	;
dsdt(	40	,1)	=	R14-R15-R17+R18-R19+R20	;
dsdt(	41	,1)	=	-R15+R16	;
dsdt(	42	,1)	=	R15-R16	;
dsdt(	43	,1)	=	R17-R18	;
dsdt(	44	,1)	=	R19-R20	;
dsdt(	45	,1)	=	R55-R33-R35+R38-R39	;
dsdt(	46	,1)	=	-R33+R34	;
dsdt(	47	,1)	=	R33-R34	;
dsdt(	48	,1)	=	-R35+R45	;
dsdt(	49	,1)	=	R35-R36	;
dsdt(	50	,1)	=	-R36+R38-R40	;
dsdt(	51	,1)	=	R36-R37	;
dsdt(	52	,1)	=	R37-R39+R43-R44	;
dsdt(	53	,1)	=	R37-R38+R41	;
dsdt(	54	,1)	=	R39-R40	;
dsdt(	55	,1)	=	R40-R41	;
dsdt(	56	,1)	=	R41-R42-R46+R47	;
dsdt(	57	,1)	=	-R42+R43-R44+R45+R47	;
dsdt(	58	,1)	=	R42-R43	;
dsdt(	59	,1)	=	R44-R45	;
dsdt(	60	,1)	=	-R46	;
```

```
dsdt(   61      ,1)     =       R46-R47         ;
dsdt(   62      ,1)     =       -R54+R34        ;

return
```
